# Four-dimensional mapping of dynamic longitudinal brain subcortical development and early learning functions in infants

**DOI:** 10.1038/s41467-023-38974-9

**Published:** 2023-06-22

**Authors:** Liangjun Chen, Ya Wang, Zhengwang Wu, Yue Shan, Tengfei Li, Sheng-Che Hung, Lei Xing, Hongtu Zhu, Li Wang, Weili Lin, Gang Li

**Affiliations:** 1 Department of Radiology and Biomedical Research Imaging Center, University of North Carolina at Chapel Hill, 130 Mason Farm Rd, Chapel Hill, NC 27599 USA; 2 Department of Biostatistics, University of North Carolina at Chapel Hill, 130 Mason Farm Rd, Chapel Hill, NC 27599 USA; 3 UNC Neuroscience Center, University of North Carolina at Chapel Hill, 116 Manning Rd, Chapel Hill, NC 27599 USA

**Keywords:** Neural patterning, Development of the nervous system

## Abstract

Brain subcortical structures are paramount in many cognitive functions and their aberrations during infancy are predisposed to various neurodevelopmental and neuropsychiatric disorders, making it highly essential to characterize the early subcortical normative growth patterns. This study investigates the volumetric development and surface area expansion of six subcortical structures and their associations with Mullen scales of early learning by leveraging 513 high-resolution longitudinal MRI scans within the first two postnatal years. Results show that (1) each subcortical structure (except for the amygdala with an approximately linear increase) undergoes rapid nonlinear volumetric growth after birth, which slows down at a structure-specific age with bilaterally similar developmental patterns; (2) Subcortical local area expansion reveals structure-specific and spatiotemporally heterogeneous patterns; (3) Positive associations between thalamus and both receptive and expressive languages and between caudate and putamen and fine motor are revealed. This study advances our understanding of the dynamic early subcortical developmental patterns.

## Abstract (web summary)

Studies of early brain development often rely on cross sectional data. Here the authors investigate volumetric development and surface area expansion of six subcortical structures using longitudinal MRI scans within the first two postnatal years.

## Introduction

During the first two postnatal years, the human brain development features a complex, dynamic, and regionally-heterogeneous process, making it critically important for later behavioral and cognitive abilities^1–5^. The recent availability of large-scale datasets of longitudinal infant brain magnetic resonance imaging (MRI) has dramatically advanced our understanding of the early development of the cerebral cortex^6^. It has been shown that the volume of the cerebral cortex increases by about 116%, and cortical surface area expands by almost 200%^7–9^ in spatiotemporally heterogeneous manners during the first two postnatal years^10^. However, our knowledge of the early spatiotemporal dynamics of deep subcortical structures and the relationship between their development and behaviors (and cognition) are still scarce, due to the huge challenges in the acquisition and analysis of infant brain MR images (typically with low tissue contrast and dynamic appearance). Of note, the subcortical structures, as the intimate working partners of the cerebral cortex, undertake a plethora of complex functions^11–16^ and have been reliably related to many neurodevelopmental and psychiatric disorders^17,18^.

Existing few studies of early subcortical development typically employed small imaging datasets with very narrow age ranges or very spare time points^8,19–23^, thus cannot fully characterize the detailed dynamic, nonlinear, and complex spatiotemporal patterns of subcortical structures. Importantly, studies exploring the relationship between subcortical development and early infant behaviors and cognition are still lacking. A representative study on the total volume of the subcortical structures at three time points, i.e., 0-month, 12-month, and 24-month, revealed that during the first year, the hippocampus showed a relatively slow volumetric development compared to other subcortical structures, and the growth of the putamen decreased faster than other structures thereafter^19^. One recent study focusing on the region-specific changes within each subcortical structure during age 5–25 years revealed regionally heterochronous developmental patterns^24^. For instance, the subregions of the thalamus, which are tightly interconnected with the cerebral cortex (e.g., superior parietal and rostral prefrontal association cortices), display significant contraction during the age range examined, while other subregions, linked to caudolateral prefrontal, inferior temporal, occipital cortices, etc., keep expanding^24^. Therefore, it is highly necessary to characterize the detailed spatiotemporal developmental patterns of each subcortical structure and discover their associations with behavioral and cognitive development during early ages.

To fill this critical knowledge gap, in this study, we unprecedentedly and comprehensively investigate the dynamic early development of the thalamus, caudate, putamen, pallidum, hippocampus, and amygdala during infancy, by leveraging 513 high-resolution longitudinal MRI scans densely covering the first two postnatal years from 231 typically developing infant subjects from the UNC/UMN Baby Connectome Project^25^. Specifically, we explore both the gross volumetric developmental patterns and spatiotemporally-detailed surface expansion maps of each subcortical structure and investigate their relationships with 6 Mullen scores^26^ (e.g., the gross motor (GM), fine motor (FM), visual reception (VR), receptive language (RL), expressive language (EL), and early learning composite (ELC)). To this end, state-of-the-art infant-dedicated deep-learning-based techniques^27,28^ are employed for MR image processing and subcortical segmentation. This study greatly advances our understanding of the dynamic, fine-grained, heterogeneous longitudinal developmental patterns of the subcortical structures during infancy.

## Results

This study included 513 longitudinal structural MRI scans from 231 infants (126 females, 105 males) in the first two postnatal years. The gross volumetric development of each subcortical structure was first investigated, and then the 4D (spatiotemporal) local surface area expansion map of each subcortical structure was delineated. Finally, the association analyses between volumetric development and surface area expansion of subcortical structures and 6 Mullen standardized t-scores were conducted.

### Trajectories of early subcortical volumetric development

**Fig. 1:**
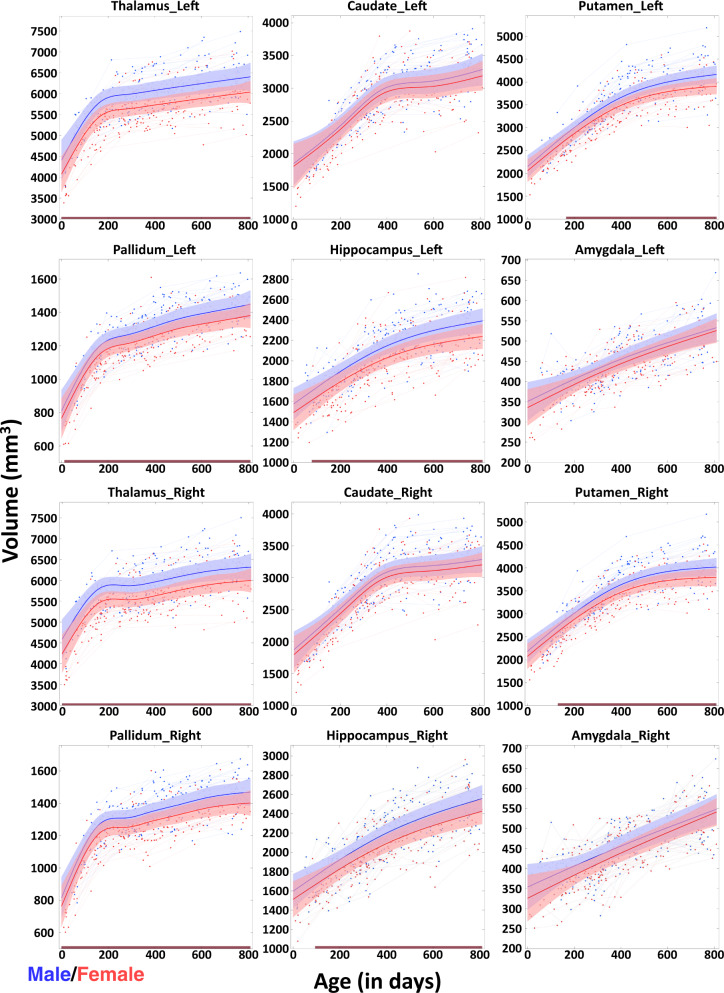
Longitudinal volumetric developmental trajectories of different subcortical structures. Plots show both the individual-level (thin lines) and fitted population-level (bold curves) volume developmental trajectories of the bilateral thalamus, caudate, putamen, pallidum, hippocampus, and amygdala structures. The shaded ribbon around each curve denotes 95% confidence intervals with males in blue and females in red.

**Fig. 2:**
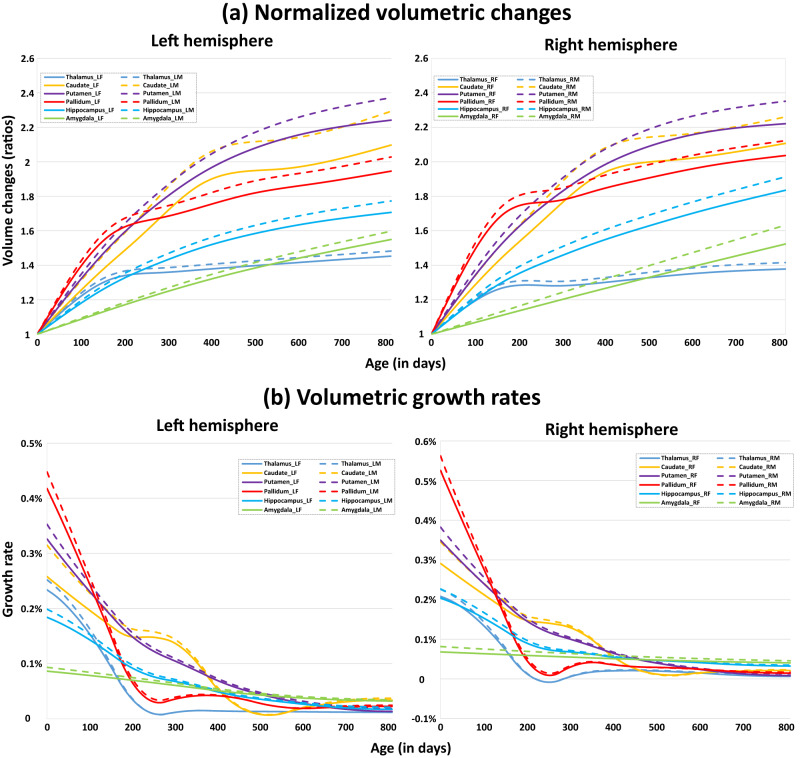
Normalized volumetric developmental curves and the volumetric growth rate curves. **a** The normalized volumetric developmental curves, compared to the volumes at term birth; **b** The curves of the volumetric growth rates for each subcortical structure. LM Left hemisphere for males, LF Left hemisphere for females, RM Right hemisphere for males, RF Right hemisphere for females.

Figure 1 delineates the longitudinal volumetric developmental trajectories of each subcortical structure (left and right) for each subject and the general additive mixed models (GAMM) fitted population-level developmental trajectories, adjusted for intracranial volume (ICV). The absolute volumetric developmental trajectories without controlling ICV are shown in Supplementary Fig. S5. The curves of the normalized volumetric ratio (compared to the volume at birth) and the growth rates of subcortical volumes are delineated in Fig. 2a and b, respectively. In Tables 1 and 2, the volumetric growth rates of all subcortical structures during different age ranges (0–3 months, 3–6 months, 6–9 months, 9–12 months, 12–18 months, 18–24 months, 0–6 months, 0–12 months, and 0–24 months) are detailed and the age ranges with statistically significant volume changes are indicated in bold (*p* < 0.05 after Bonferroni Correction). For the corresponding *P*-values, please see Supplementary Tables S5 and S6. Combining Fig. 1, Tables 1 and 2, we can find that the gross volumes of subcortical structures (except for the amygdala) increase rapidly within the first several months of age, and then the volume enlargement gradually slows down starting from different ages. Different from other structures with nonlinear development, the amygdala exhibits an approximately linear increase with a relatively low growth rate during the first two postnatal years. Of note, most structures present statistically significant volumetric growth during the age range examined, while only the thalamus within the age range from 6M to 12M (M: month) is not statistically significant. Besides, the thalamus, putamen, pallidum, and hippocampus gradually exhibit significantly larger volumes in males than in females after birth (*p* < 0.05). Moreover, each subcortical structure shows a bilaterally relatively similar developmental pattern. From Fig. 2a, we observe that nearly all subcortical structures keep rapidly growing followed by slow increases relative to their initial volume at birth. From Fig. 2b, we find the growth rates of the pallidum and putamen are high initially after birth, while the amygdala exhibits the lowest growth rates. The growth rates of the pallidum and thalamus decrease more rapidly after birth, compared to the growth rates of other structures. Finally, the preliminary results of the asymmetry index trajectories of each subcortical structure are shown in Supplementary Fig. S4. For further details, please see Supplementary Information 5. Asymmetry Index Trajectories of Subcortical Volumes.

**Table 1:** Volumetric growth rates of the left subcortical structures at different developmental stages during the first two years of age

Age range	Thalamus_L	Caudate_L	Putamen_L	Pallidum_L	Hippocampus_L	Amygdala_L
	(Male/Female)	(Male/Female)	(Male/Female)	(Male/Female)	(Male/Female)	(Male/Female)
	(%)	(%)	(%)	(%)	(%)	(%)
0M-3M	**20.1/21.6**	**21.4/21.5**	**24.0/24.2**	**36.7/38.7**	**12.5/12.6**	**7.8/8.4**
3M-6M	**10.6/11.3**	**18.1/18.2**	**18.5/18.6**	**17.7/18.3**	**10.6/10.7**	**7.1/7.6**
6M-9M	2.1/2.2	**16.3/16.4**	**14.4/14.4**	**4.7/4.6**	**8.9/9.0**	**6.4/6.9**
9M-12M	1.2/1.2	**11.9/11.9**	**10.6/10.4**	**3.8/3.7**	**7.0/7.0**	**5.6/6.0**
12M-18M	**2.5/2.6**	**6.2/5.8**	**11.5/10.9**	**6.5/6.3**	**8.4/8.1**	**8.7/9.3**
18M-24M	**2.3/2.3**	**4.8/4.3**	**5.1/4.1**	**4.3/3.9**	**4.6/4.0**	**7.2/7.7**
0M-6M	**32.8/35.3**	**43.3/43.7**	**47.0/47.2**	**60.9/64.1**	**24.4/24.7**	**15.4/16.6**
0M-12M	**37.2/39.9**	**86.5/87.3**	**86.0/85.9**	**74.9/77.9**	**45.1/45.3**	**29.7/32.1**
0M-24M	**43.9/46.8**	**107.6/106.6**	**117.9/114.5**	**94.3/96.6**	**64.5/63.3**	**51.1/55.6**

Values in bold indicate statistically significant changes with *p* < 0.05 after Bonferroni Correction.

*M* Month.

**Table 2:** Volumetric growth rates of the right subcortical structures at different developmental stages during the first two years of age

Age range	Thalamus_R	Caudate_R	Putamen_R	Pallidum_R	Hippocampus_R	Amygdala_R
	(Male/Female)	(Male/Female)	(Male/Female)	(Male/Female)	(Male/Female)	(Male/Female)
	(%)	(%)	(%)	(%)	(%)	(%)
0M-3M	**17.7/19.2**	**23.2/24.3**	**23.8/24.4**	**47.2/48.1**	**12.3/12.6**	**6.1/7.3**
3M-6M	**8.6/9.2**	**18.6/19.3**	**18.3/18.7**	**19.6/19.5**	**10.6/10.8**	**5.7/6.8**
6M-9M	0.2/0.3	**15.6/16.1**	**14.1/14.3**	**3.0/2.3**	**9.1/9.2**	**5.5/6.5**
9M-12M	0.9/1.0	**11.0/11.3**	**10.2/10.2**	**3.7/3.1**	**7.4/7.5**	**5.2/6.0**
12M-18M	**3.7/4.0**	**6.3/6.3**	**10.5/10.1**	**5.9/5.7**	**10.0/9.9**	**9.1/10.6**
18M-24M	**2.3/2.5**	**3.1/2.9**	**3.7/2.9**	**3.7/4.9**	**6.8/6.5**	**8.7/10.0**
0M-6M	**27.8/30.2**	**46.1/48.3**	**46.4/47.7**	**76.0/77.0**	**24.2/24.8**	**12.2/14.7**
0M-12M	**29.2/31.9**	**87.4/91.6**	**84.2/86.0**	**88.0/86.6**	**45.6/46.6**	**24.5/29.4**
0M-24M	**37.1/40.7**	**105.5/109.6**	**111.0/110.8**	**106.4/107.0**	**70.9/71.5**	**47.7/57.5**

Values in bold indicate statistically significant changes with *p* < 0.05 after Bonferroni Correction.

*M* Month.

### 4D surface area expansion maps of subcortical structures

**Fig. 3:**
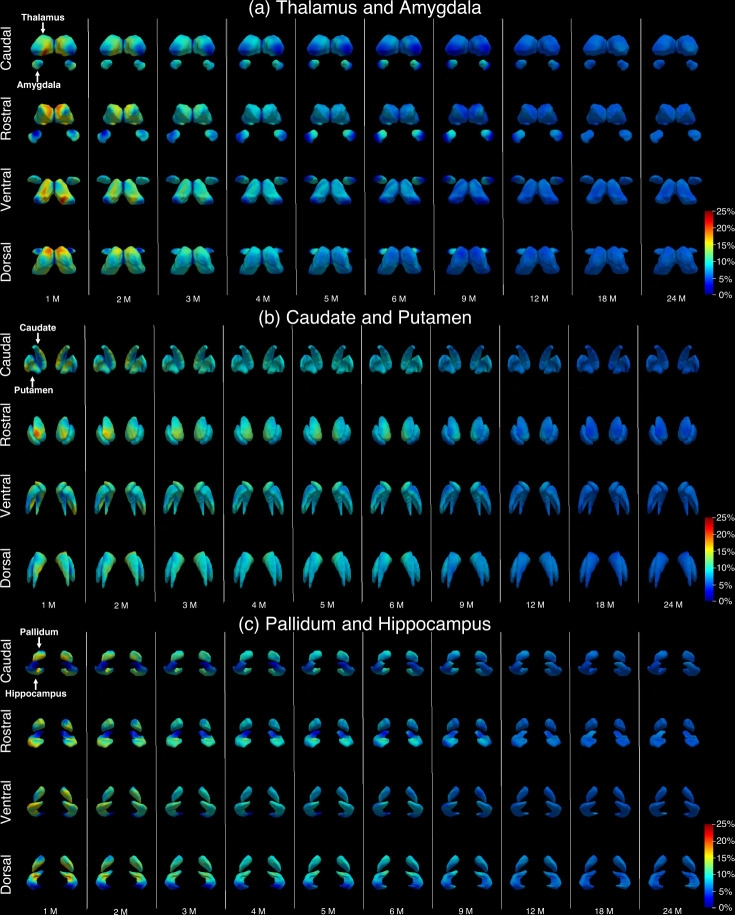
4D monthly growth rate maps of surface area. **a** Thalamus and amygdala, **b** caudate and putamen, **c** pallidum and hippocampus. M Month.

**Fig. 4:**
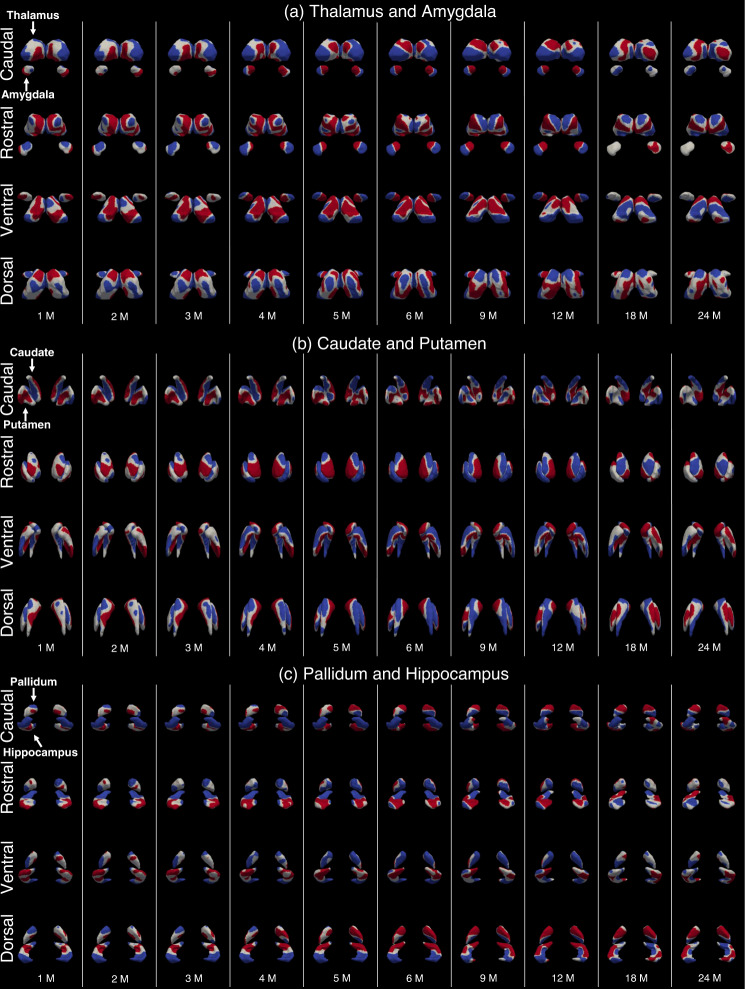
Statistically significant high-growth and low-growth regions (*p* < 0.05, FDR adjusted) at different ages. **a** Thalamus and amygdala, **b** caudate and putamen, **c** pallidum and hippocampus. Red colors denote high-growth regions. Blue colors denote low-growth regions. M Month.

Figure 3 shows the 4D expansion (growth rate) maps of the surface area of each subcortical structure, which shows that the local growth rates vary significantly within each subcortical structure during infancy. Overall, the area expansion of each subcortical structure exhibits distinct, age-dependent, region-specific, and bilaterally quasisymmetric patterns, with the growth rates initially being rapid and gradually slowing down. The statistically significant high-growth and low-growth regions of each subcortical structure are illustrated in Fig. 4. Specifically, the high-growth and low-growth regions were first extracted based on the growth rate at each month with the threshold of top and bottom 20% vertices, respectively. Then, a t-test (as shown in the Supplementary Information 2. Materials and Methods) was performed to test if the growth rate at each vertex is significantly larger or smaller than the median of growth rates across all vertices. After correction for multiple comparisons using Benjamini–Hochberg’s false discovery rate (FDR), the vertices with statistically significant (*p* < 0.05, FDR adjusted) high-growth and low-growth rates were illustrated in Fig. 4 in red and blue colors, respectively.

For the thalamus, the bilateral pulvinars expand significantly lower than other regions during the first year and gradually becomes a high-growth region within the second year. The bilateral anterior facets (anterior nuclei) are the high-growth regions from birth to 5 months of age and become low-growth regions thereafter. The lateral dorsal and posterior facets turn to high-growth regions after 9 months of age. The posterior dorsomedial facets (medial dorsal nuclei) are high-growth regions within the first year and gradually turn into low-growth regions in the second year. The bilateral anterolateral facets (ventral anterior and lateral nuclei) belong to low-growth regions from birth to 5 months of age and become high-growth regions with advancing age.

For the amygdala, the high growth of the bilateral medial rostral regions mainly appears from 4 to 12 months of age. The high growth of the bilateral lateral facets mainly emerges during the first 3 months of age. In the second year, the facets adjacent to the hippocampus expand slowly, while the other facets grow rapidly.

For the caudate, the dorsal medial caudate head and body are high-growth regions from birth to 6 months of age and the dorsal medial caudate head is the main high-growth region thereafter. The dorsal low-growth regions gradually increase from the middle part and extend to the caudate tail during the first 18 months. The ventral caudate exhibits low growth from birth to 5 months of age, and the ventral caudate body and head gradually become high-growth regions in the remaining age range examined.

For the putamen, the bilateral medial facets adjacent to the pallidum are high-growth regions until around 6 months of age and become low-growth regions with advancing age. The lateral facets of the putamen exhibit high growth from 4 to 12 months of age. Moreover, the facets close to caudate are low-growth regions until 6 months of age, and then turn to high-growth regions.

For the pallidum, from birth to 4 months of age, the ventral facets away from the caudate are high-growth regions and change into low-growth regions thereafter. The bilateral dorsal facets adjacent to the caudate become high-growth regions after 5 months of age, which exhibit consistent expansion patterns with the neighboring thalamus and caudate facets.

For the hippocampus, the hippocampus tails grow slowly, while the hippocampus heads exhibit a high expansion rate during the first two postnatal years. The lateral facets of the hippocampus body become high-growth regions after 5 months of age.

In Supplementary Movies, Movie S1 illustrates the dynamic surface area expansion rate map of the infant subcortical structures from birth to 24 months of age. Movie S2 also exhibits the dynamic infant subcortical surface area expansion rate map in 4 fixed views (same as Fig. 3). For further details, please see SI: Movies.

We also tested the sex effect of the rate of surface area expansion for each subcortical structure but didn’t find any sex difference.

### Significant associations between volumetric development and area expansion of subcortical structures with Mullen scales

#### Volumetric development associated with Mullen scales

In Table 3, the association analysis results (standardized coefficients (*β*) with FDR adjusted *P*-values (*p*)) between the relative volumes of each subcortical structure (ratio of the subcortical volume to the intracranial volume) and each of the 5 Mullen standardized t-scores and the early learning composite values are presented. After FDR correction, it is discovered that the volume ratios of the caudate (*β* = 1.95; *p* = 0.013) and putamen (*β* = 2.13; *p* = 0.012) are positively associated with the fine motor. Meanwhile, the volume ratio of the thalamus is positively associated with receptive language (*β* = 3.86; *p* = 0.004) and expressive language (*β* = 3.03; *p* = 0.013), respectively. To further testify the above-discovered associations, the identified subcortical volume ratios were put together to fit the Mullen scores using a multiple regression model (Eq. (5) in the Materials and Methods). Results illustrate that the multiple regression model in fitting the fine motor based on the volume ratios of caudate and putamen are significant with *p* = 0.0007, suggesting the positive correlations between the development of the caudate and putamen and the fine motor functions. Similarly, the volume ratio of thalamus-based fitting also exhibits statistical significance for both receptive language (*p* = 0.0001) and expressive language (*p* = 0.0011), confirming the significant positive associations between the development of the thalamus and both receptive and expressive language functions. For more details of multiple regression model-based results, please see Supplementary Information 4. Detailed results for multiple regression.

**Table 3:** Standardized coefficients/*P*-values (FDR-adjusted) between Mullen scores and subcortical structures during the first 2 years of age

(Coef/*P*-value)	GM	VR	FM	RL	EL	ELC
Thalamus	0.295/0.879	1.494/0.542	−2.186/0.177	**3.959/0.004**	**3.093/0.013**	3.022/0.160
Caudate	0.298/0.864	−0.683/0.736	**1.911/0.020**	−0.790/0.670	−0.077/0.899	0.161/0.841
Putamen	−0.168/0.822	−1.236/0.294	**2.118/0.013**	−1.248/0.294	−0.546/0.742	−0.410/0.814
Pallidum	0.692/0.858	0.379/0.833	0.790/0.770	0.642/0.813	1.451/0.406	1.415/0.709
Hippocampus	−0.466/0.888	−0.461/0.845	0.720/0.751	0.308/0.893	0.378/0.866	0.431/0.875
Amygdala	−0.578/0.732	0.525/0.801	−0.947/0.601	0.154/0.862	-0.139/0.848	−0.175/0.825

Values in bold indicate statistically significant associations with *p* < 0.05 after FDR correction.

#### Area expansion associated with Mullen scales

**Fig. 5:**
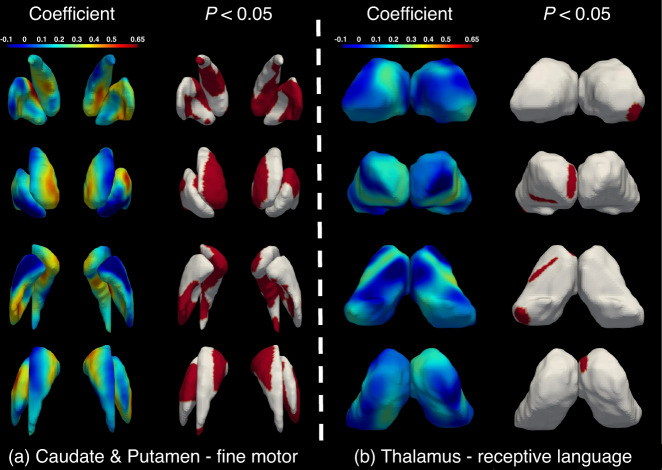
Coefficient values and statistically significant areas associated with behaviors after FDR correction (*p* < 0.05). **a** Caudate & putamen with fine motor, **b** thalamus with receptive language. Red colors denote positive associations.

Based on the above-discovered associations between the gross volumetric development of each subcortical structure and Mullen scales, we further performed the association analysis between the surface area expansion of significant subcortical structures and the Mullen t-scores (thalamus-receptive language, thalamus-expressive language, and caudate- & putamen-fine motor). The areal association results (standardized coefficients with significantly related subregions (FDR-adjusted *P*-values)) were illustrated in Fig. 5. We can observe that the caudate and putamen exhibit significant positive associations with fine motor scores in multiple subregions. Specifically, the bilateral medial caudate head and both medial and lateral dorsal regions of the putamen are positively associated with fine motor scores, which is highly consistent with the high-growth regions of the caudate and putamen between 4 and 12 months of age. Besides, several subregions of the thalamus (the right pulvinar, medial anterior dorsal nuclei, and ventral anterior and lateral nuclei) are excavated to associate with receptive language, while no thalamic subregion exhibits significant association with expressive language.

## Discussion

In this paper, we unveiled the longitudinally continuous volumetric developmental trajectories and detailed dynamic area expansion patterns of six subcortical structures, including the thalamus, caudate, putamen, pallidum, hippocampus, and amygdala, within the first two postnatal years. Our results demonstrate that the volumes of most subcortical structures exhibit highly remarkable growth during the first several postnatal months followed by a sluggish increase. Meanwhile, most subcortical structures (except for the caudate and amygdala) exhibit statistically significant sex differences, with males consistently having larger volumes than females. Each subcortical structure presents its own distinct and spatiotemporally heterogeneous surface area expansion with advancing age. Both the volumetric development and the surface area expansion of the caudate and putamen are significantly positively correlated with the fine motor scale. Furthermore, the thalamus volumetric development is positively associated with receptive and expressive languages. Meanwhile, the area expansion of several subregions of the thalamus is associated with receptive language. These findings provide valuable information about the relationship between subcortical structural development and early learning functions.

In the first two postnatal years, we find no volumetric peak of any of the six subcortical structures, which is in line with the findings from previous studies on older children (≥ 3 years of age) showing that the volumes of the caudate, putamen, and pallidum firstly peak in childhood, while the volumes of the thalamus, hippocampus, and amygdala peak later^24,29–34^. Except for the amygdala, which develops almost linearly during infancy, the volumes of the remaining five subcortical structures increase rapidly during the first few months, and then their growth slows down starting at different ages thereafter. A previous study of the whole subcortical volume with very sparse data points (1 month, 12 months, and 24 months of age)^8^ reported a similar nonlinear volumetric enlargement with a striking ascension followed by a sluggish uprise during the first two postnatal years, which is generally consistent with our results. Another study^19^ calculated the volumetric change of each subcortical structure at the same 3 time points, strengthening our results that subcortical structures develop more dramatically within the first year than the second year, while our results detailed the developmental patterns and turning time points of each subcortical structure.

The observed differential nonlinear volumetric developmental patterns suggest that the developmental processes are structure specific. The heterochronous developmental pattern is also observed within each subcortical structure. Several factors may contribute to the volumetric growth and surface expansion of subcortical structures. Like principal neurons in the cerebral cortex, neurons in the subcortical structures are also different from progenitor cells in the ventricular zone^35,36^. In general, the rapid volumetric growth at the early developmental stage is mainly contributed by neurogenesis from progenitor cells. In addition, later neuronal maturation, such as enlargement in cell size and elaboration of neuronal processes, and the development of non-neuronal cells can also contribute to volumetric growth^37^. Speculatively, the volumetric increase of the hippocampus in early postnatal years may be contributed by the proliferation and migration of the pyramidal cells, which are the principal neurons of hippocampal subdivisions (CA1, CA2, and CA3)^38,39^. Besides, the development of hippocampal interneurons (e.g., basket cells, bistratified cells, and Oriens-lacunosum moleculare cells), which serve as local circuit neurons, could also accelerate the rapid growth of hippocampal volumes^38,39^. Unlike the other five subcortical structures, the amygdala exhibits linear volumetric growth in the first two years of infancy and continues growing until around 10 years of age^29^. A stereological study of the postnatal amygdala in the rat brain showed that the continuous volumetric growth of the amygdala correlates to the increase in the numbers of oligodendrocytes^40–45^. A similar increase in oligodendrocytes and subsequent myelination in the human brain may contribute to the volumetric growth of the amygdala and other subcortical structures, especially in the sluggish growth phase. However, we could not rule out the possible contribution of late-phase neurogenesis in some subcortical structures without knowing the birth time of neurons in these structures.

Besides, the developmental trajectories of subcortical structures are sexually dimorphic with males consistently having larger absolute volumes than females, which is also in line with the results of recent studies on old children’s subcortical development^29^. This may be partly attributed to the difference in sexual hormones^40^. The sex differences of subcortical volumes may provide a structural basis of reactivity to distinct surroundings^46^.

In our study, each subcortical structure shows profound region-specific expansion during infancy, i.e., each subcortical subregion exhibits different age-dependent significantly fast or relatively sluggish outspread. Such regionally-heterogeneous developmental patterns within each subcortical structure may be partly related to the development of the associated cerebral cortical regions or other subcortical structures through anatomical or functional connectivity.

The region overlapping with the medial dorsal nuclei in the thalamus, which is confirmed to connect to the medial prefrontal cortex (MPFC) and orbitofrontal cortex (OFC)^24,47,48^, shows high expansion within the first postnatal year and gradually becomes a low-expansion region in the second postnatal year in our results. Our previous study on longitudinal cortical surface expansion within the first two postnatal years has confirmed that the OFC displays dramatic expansion (80%) during the first year of age followed by a relatively slow expansion of 20% in the second year^9^, which is generally in line with our results in this work on the subcortical development. Meanwhile, the cortical thickness of the medial OFC peaks very first at around 12 months of age^6^. In addition, the high-growth regions in the caudate head, medial caudal facets of the putamen, and dorsal facets of the pallidum during the first postnatal year might project to MPFC^24,47,48^. Our previous study^6^ also demonstrates that the cortical thickness of the MPFC develops rapidly within the first year of life. This high consistency and synchronization with the development of the cerebral cortex reinforce the reliability of our results.

The hippocampus head exhibits a high growth rate during the first 6 months of age, whereas the development of the hippocampus tail is stagnant, which suggests a developmental gradient along the long-axis from head to tail. Previous functional connectivity studies of the hippocampus implicated that the hippocampus head shows preferential functional connectivity with a large number of brain regions, i.e., the temporal cortex, frontal cortex, parietal cortex, occipital cortex, and limbic region, compared to the hippocampus tail and body^49^. The early rapid expansion of the hippocampus head could be explained by the complicated anatomical and functional relevance between the hippocampus head and multiple cortical regions. Moreover, from 9 months of age, the high growth rate of the lateral facets of the hippocampus body, in addition to the low growth rate of medial facets, suggests a width increase in the short-axis with a direction from medial to lateral. Such rapidly growing lateral facets are highly consistent with the hippocampal cornu ammonis (CA1) subregion, which is generally linked to the retrieval of memory^50^. Therefore, we speculate that this rapidly developing CA1 subregion may be the neural foundation for the ability to retrieve knowledge from recent memory as they are also confirmed to appear and improve during the same age range^7,19,50–53^.

The amygdala preferentially connects to the dorsal striatum (i.e., caudate and putamen) and MPFC^54–56^. Specifically, the area expansion of the medial amygdala is low, corresponding to the slow expansion of the caudate and putamen during the first several months, as shown in Fig. 4. On the contrary, the lateral amygdala grows rapidly, which could coordinate with the early rapid development of the MPFC during the same age period^6^. Our finding of the longitudinal regional development of the amygdala also shows remarkable consistency with such bipartite partition^57^, that from 4 to 12 months of age, the discovered high-growth and low-growth subregions highly match up to the medial and lateral amygdala, respectively, further strengthening the meaningfulness of our results. In conclusion, the early surface area expansion in each subcortical structure could be partly explained by the complicated anatomical connections.

The volumetric developmental patterns of the subcortical structures are closely linked to behavioral development during infancy. Specifically, the volumetric development of the caudate and putamen are significantly associated with the fine motor, revealing that the caudate and putamen may directly or indirectly influence the establishment and maturation of the fine motor function. In ref. ^58^, the authors also confirmed the association between the reduced volume of the caudate nuclei and the impaired fine movement coordination in patients with hippocampal atrophy aged from 9 to 25 years of age. In addition, a neonatal animal study^59^ demonstrated that the number of the medium-spiny projection neurons, the principal neurons which account for around 95% of all the neurons in the striatum, is significantly associated with the long-term fine motor capability on the staircase test. Taken together, the striatum should undertake key elements in the establishment of fine motor. Moreover, the surface area expansion results in our research suggest that the caudate head and dorsal regions of the putamen are significantly related to fine motor, which specifies the fine motor-related location at the vertex level and suggests the importance of these regions in the establishment of fine motor. The basal ganglia ischaemic stroke patients aged ≥ 6 years with lesions on the caudate head were confirmed to have fine motor disorders and changes in handedness^60^, which support our results. The possible explanation may be related to the anatomical and/or functional connectivity with the cerebral cortex^60^, and further connections between different regions may confirm this.

Except for the association with fine motor, our correlation results also reveal that the development of the thalamic volume is significantly positively correlated with expressive and receptive languages. A plethora of research has confirmed the tight relationship between the thalamus and language in normal and abnormal subjects^61–63^. For example, the volumes of bilateral thalami are significantly correlated with language-related cognitive scores in individuals at high genetic risk for schizophrenia during 16–30 years of age, which is consistent with our results. The thalamic-cortical pathways may contribute to this relationship and distinct thalamic nuclei exhibit synergistic roles in different pathways associated with language. A diffusion-weighted imaging fiber tracking study^64^ demonstrates a direct connection between the Broca’s area and the ventral anterior nucleus of the thalamus, which supports the relationship between the volumetric development and expressive language. The view of ref. ^65^ also considers that the process for extracting auditory source and identity information depends on the neuroanatomical connections between the thalamus and cortex, again sustaining our discovery of the association between the thalamus and receptive language. Moreover, the surface area expansion at the vertex level suggests that the right pulvinar is significantly related to the receptive language. In detail, the medial geniculate body of the thalamus (located at the posterolateral surface of the pulvinar), as the principal nucleus receiving ascending auditory information^66^, may contribute to such significant association. Further research may confirm our results.

A few limitations of the present study should be noted. First, almost 40% of the subjects have only one scan, which could potentially influence the longitudinal consistency and accuracy. Second, the subject number is imbalanced across different ages, e.g., fewer scans within the first two and last several months, except for the 24-month with more than 30 scans, which could also negatively affect the accuracy of the longitudinal analysis. Third, although a state-of-the-art infant dedicated learning-based subcortical segmentation method was performed and achieved overall high accuracy, the manually-corrected segmentations of the pallidum and amygdala still suffer from relatively low reliability in the first few months due to the low tissue contrast. Compared to the thalamus and putamen, the pallidum is more indistinctive with the surrounding white matter, even in adult MRI^67^. The amygdala during infancy is too small to be well labeled, making manual delineation very challenging^28^. Fourth, we focused on the dynamic subcortical development during infancy, due to relatively limited data after 2 years of age in the BCP dataset. A more rigorous investigation including neonates, infants, toddlers, preschoolers, and children data acquired with similar scanners and imaging protocols would be helpful to draw conclusions about potential turning points of trajectories of the subcortical development. This is an important topic for our future work.

In summary, our study has two major contributions. First, we have unprecedentedly charted the continuous volumetric developmental trajectories and region-specific area expansion of six subcortical structures during the first two postnatal years. Second, our study has unveiled the significant positive relationship between the early volumetric development and area expansion of each subcortical structure with the behavior scores. These discoveries fill critical knowledge gaps and provide a fundamental reference and insight for future studies regarding both normal and abnormal subcortical development.

## Methods

### Participants

**Fig. 6:**
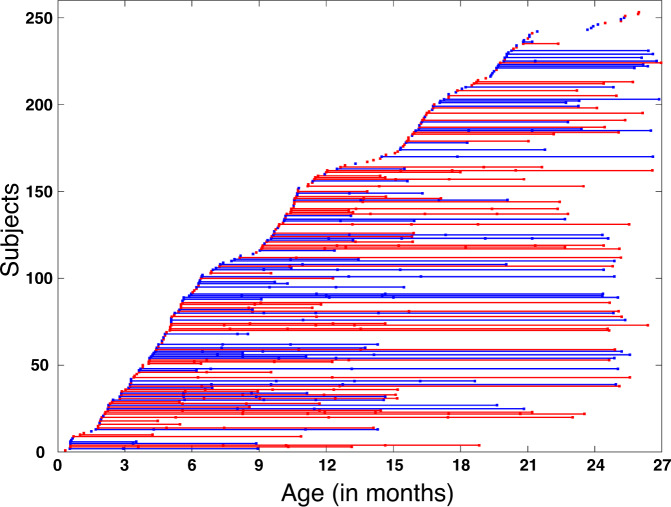
Distribution of MRI scans. Each point represents a scan at its scanned age shown in the x-axis. Each horizontal line represents one subject, with males in blue and females in red.

This study was approved by the Institutional Review Board at the University of North Carolina (UNC) at Chapel Hill, School of Medicine. All images used in this study were from the UNC/UMN Baby Connectome Project (BCP)^25^, which recruited typically developing children from birth to 5 years of age. The participants were recruited from board communities to ensure that the sample approximates the racial/ethnic and socioeconomic diversity of the US census. In total, 513 longitudinal MRI scans from 231 subjects (including 105 males with 233 scans and 126 females with 280 scans) up to 27 months of age were included in this study to investigate subcortical development during infancy. In the dataset, 45% of infants had 2 or more scans, and 12% of infants had 3 or more scans. The distribution of longitudinal scans across the study age span is illustrated in Fig. 6. For further details, please see Supplementary Table S1 and Supplementary Fig. S1.

### Image acquisition & collection of Mullen scores

All BCP MR images were acquired at two sites located at the Biomedical Research Imaging Center (BRIC) at the University of North Carolina at Chapel Hill (UNC site) and the Center for Magnetic Resonance Research (CMRR) at the University of Minnesota (UMN site), respectively. To mitigate the site difference, two 3T Siemens Prisma MRI scanners were exploited with the same Siemens 32 channel head coils and imaging protocols. Meanwhile, the images acquired at the two sites passed the same imaging procedures and quality control criteria (i.e., excessive motion, insufficient coverage, and ghosting^25^). In detail, all infants were scanned when they were naturally sleeping, and their heads were secured in a vacuum fixation device with ear protection. T1w and T2w images have a spatial resolution of 0.8 × 0.8 × 0.8 mm^3^ and were acquired with 208 sagittal slices using the following parameters, respectively: TR/TE = 2400/2.24 ms and TR/TE = 3200/564 ms.

In total, 702 scans from 306 subjects within 26 months of age were acquired. All scans were checked through visual inspection-based quality control to exclude the scans with motion artifacts, insufficient coverage, and ghosting, and 633 scans passed the quality control. 564 out of 633 scans had both T1w and T2w images. For more details on the BCP dataset and the MR images used in this work, please see Supplementary Information 2. Materials and Methods.

We collected the age-normalized t-scores, age equivalent scores, and percentile rankings as Mullen scores for gross motor, fine motor, visual reception, receptive language, and expressive language, respectively. Then, the values of early learning composite were aggregated from the scores of fine motor, visual reception, receptive language, and expressive language. The Mullen scores were implemented at every behavioral visit between 3 and 60 months of age and each assessment took between 20 and 45 mins, varying with the age of the child. Further details could be found in ref. ^25^.

### Image processing

All images were then preprocessed using the infant brain extraction and analysis toolbox (iBEAT V2.0 Cloud) (http://www.ibeat.cloud/)^27^. Specifically, the N3 method^68^ was first performed on all images for intensity inhomogeneity correction. Then a learning-based method was used for skull-stripping^69^. Subsequently, for each subject, the T2w image was linearly aligned onto the corresponding T1w image using FLIRT in FSL^70^. Both T1w and T2w images were fed into our developed convolutional neural networks for infant brain subcortical segmentation^28^. Each subcortical segmentation map was further manually checked and corrected by one expert (with more than 4 years of experience in infant brain MR image segmentation) to ensure the segmentation accuracy and consistency. In detail, for each suspicious label, we localized it in the 3 canonical views, i.e., axial, sagittal, and coronal views, at first. Then, we ascertained its correct label based on the two images (T1w and T2w images) by considering the 3 views together. The holes and the bulges were also fixed according to the surface rendering. Finally, 513 out of 564 scans survived in the manual check. For the segmentation results, please see Supplementary Fig. S2, Supplementary Tables S2 and S3. The following subcortical structures were extracted, including the thalamus, caudate, putamen, pallidum, hippocampus, and amygdala in each hemisphere. The total volume of each subcortical structure over two hemispheres was calculated.

For longitudinal analysis of spatially-detailed subcortical developmental patterns, we first constructed a 4D infant brain volumetric atlas^71^ based on the BCP dataset using the state-of-the-art SyGN template construction technique from ANTs^72^, to include densely sampled time points (i.e., 0, 1, 2, 3, 4, 5, 6, 7, 8, 9, 10, 11, 12, 15, 18, 21, and 24 months of age). During the atlas construction procedure, we established two sets of voxel-wise anatomical correspondences, i.e., the correspondences across atlases at different time points and the correspondences between each age-specific atlas and the age-matched individual scans. Then, to achieve more accurate registration, it is better to regard the small-size image with relatively smooth shapes as the moving image, while the large-size image containing complex shapes is the target image. Thus, we manually delineated the 12 subcortical structures (left and right) using ITK-SNAP^73^ and accordingly reconstructed surface mesh representations of each subcortical structure for the 0-month atlas as the initial reference surfaces. Next, we warped the surface mesh representations of the subcortical structures from the 0-month atlas to individual scans by exploiting both the established correspondences across age-specific atlases and established correspondences between the age-specific atlases and individual scans following an age-increasing manner. For the steps of the subcortical surfaces mapping and registration accuracy, please see Supplementary Fig. S3 and Supplementary Table S4, respectively. The obtained vertex-to-vertex corresponding subcortical surface representations for each individual scan enabled us to compare vertex-wise features across different scans at different time points. Finally, we calculated the local surface area of each vertex on each individual subcortical surface to fit the vertex-wise developmental trajectories.

### Statistical modeling

We utilized the general additive mixed models (GAMM) to fit the developmental trajectories of the total volume and each vertex’s surface area of each subcortical structure. The non-parametric GAMM was adopted, which has been shown to be one of the best fit models for early brain developmental trajectories^6^, compared with parametric models. Specifically, for N subjects, the following two models were respectively used in our model fittings for the scenarios with or without considering sex:

1 $${y}_{i}(t)={s}_{i}+{T}_{i}(t)+{{{{{{{\rm{f}}}}}}}}(t)+\Delta (t)\cdot {g}_{i}+{\alpha }_{i}+{e}_{i}(t)$$

2 $${y}_{i}(t)={s}_{i}+{T}_{i}(t)+{{{{{{{\rm{f}}}}}}}}(t)+{\alpha }_{i}+{e}_{i}(t)$$

where *s*_*i*_ is the site indicator of subject *i* to mitigate the site differences of the samples respectively acquired from UNC and UMN sites, *g*_*i*_ denotes the sex, *α*_*i*_ represents the random intercept effect, f(*t*) and Δ(*t*) are two non-parametric functions^74^ fitted with the cubic splines (with the dimensions of the bases used to represent the smooth term setting), and *e*_*i*_(*t*) and *T*_*i*_(*t*) respectively denotes the random Gaussian noise and ICV for the *i*^*th*^ subject at time *t*, which are assumed to be independent and identically distributed for *i* = 1, 2, …, *N* and *t* = 0, 1, …, 810 (in days). For further details, please see Supplementary Information 2. Materials and Methods.

For the analysis of structural volumetric development (Fig. 1), all smoothing parameters were selected by maximizing the marginal likelihood (ML). For each subcortical structure, we respectively took the volume of the left side and right side and fitted their developmental trajectory using the model (Eq. (1)) with respect to sex. We then took the first derivative of the fitted trajectory for each sex level to get the sex-specific growth rate (Fig. 2). In Tables 1 and 2, to identify the statistically significant volumetric changes of the left and right subcortical structures, we calculated the population-level volumetric growth rate between two ages *t*_1_ and *t*_2_, e.g., 0–3, 3–6, 6–9, 9–12, 12–18, 18–24, 0–6, 0–12 and 0–24 months by [**Y**(*t*_2_) − **Y**(*t*_1_)]**Y**(*t*_1_), and the *P*-values were calculated based on the Z-statistics on the growth rates of each subcortical structure (deviate from zero) ∣**Y**(*t*_2_) − **Y**(*t*_1_)∣/SD[**Y**(*t*_2_) − **Y**(*t*_1_)], where SD[**Y**(*t*_2_) − **Y**(*t*_1_)] was the standard deviation of [**Y**(*t*_2_) − **Y**(*t*_1_)] estimated from bootstrap estimation with resampling of 1000 times through the stratified bootstrap for longitudinal data^75^. The obtained *P*-values were corrected for multiple comparisons using the Bonferroni correction, and the significant changes of each subcortical structure on each specific age range (*p* < 0.05) are highlighted in bold in Tables 1 and 2.

For the analysis of the vertex-wise subcortical development, we fitted the developmental trajectory of each vertex of each structure using the model (Eq. (2)) and calculated the growth rate by taking the first derivative of the fitted curve (Fig. 3). The first derivative of the model (Eq. (2)) was used to identify the high-/low-growth regions (Fig. 4) without distinguishing sex. The t-tests were performed to test if the growth rate at each vertex is significantly larger or smaller than the median of growth rates across all vertices at each time point. After correction for multiple comparisons using Benjamini–Hochberg’s FDR adjustment, the vertices with statistically significant (*p* < 0.05, FDR adjusted) high-growth and low-growth rates were illustrated in Fig. 4 in red and blue colors, respectively, while the white regions are the areas without statistical significance. Further details are presented in Supplementary Information 2. Materials and Methods.

For the analysis between subcortical development and Mullen behavior scores (Table 3), we first calculated the logarithm of the subcortical-to-ICV (intracranial volume) ratio, and then carried out the association analyses between each subcortical volume ratio of the 6 subcortical structures and each of the 6 Mullen standardized t-scores based on linear mixed models (Eq. (3)). Specifically, 30 linear mixed models were considered. In each model, one of the 6 Mullen score subdomains, e.g., the gross motor (GM), fine motor (FM), visual reception (VR), receptive language (RL) expressive language (EL), and early learning composite (ELC) scores, was taken as the dependent variable, one of the 6 subcortical volume ratios was included as the independent variable, and the age, sex, site effect, maternal education, household income, and intracranial volume were controlled as confounding factors; meanwhile, a by-subject random intercept was included to model the longitudinal data random effect. All covariates were standardized to be mean zero and unit variance before entering the models. The Satterthwaite’s t-test for mixed models was used to test the significance of the model coefficients which were further corrected for multiple testing using the Benjamini–Hochberg’s FDR adjustment method. Finally, the identified subcortical volume ratios are put together to fit the Mullen scores using multiple regression models with random intercept (Eq. (5)). The ANOVA F-statistics was adopted to test the model significance between the Multiple regression model (Eq. (5)) and the Null model (Eq. (4)) (confounding factors only).

- Model (3): Mullen score $\sim age+sex+site+income+edu+ICV+ \log (sub\_ratio)+(1|ID)$,
- Null model (4): Mullen score ~ *age* + *sex* + *site* + *income* + *edu* + *ICV* + (1∣*ID*),
- Multiple testing model (5): Mullen score $\sim age+sex+site+income+edu+ICV+\log (A\_ratio)+\log (B\_ratio)+(1|ID)$,

where *age* is scan age, *sex* is biological sex, *site* is two sites, *income* is family income, *edu* is maternal education (with or without a graduate degree), and *ICV* is the intracranial volume.

For the area expansion analysis between caudate, putamen, and thalamus and Mullen scores (Fig. 5), we applied the same mixed linear models to generate the associations between Mullen scores and each vertex. The achieved *P*-values are further adjusted by FDR correction. Finally, we carried out the vertex-wise areal behavior analysis at each vertex from the aligned surface:

- Model (6): Mullen score ~ *age* + *sex* + *site* + *income* + *edu* + *ICV* + *area*,

where *area* is the local area at the specified vertex. The *P*-value of each vertex was corrected for multiple comparisons using Benjamini–Hochberg’s FDR adjustment method.

### Reporting summary

Further information on research design is available in the Nature Portfolio Reporting Summary linked to this article.

## Supplementary information

Supplementary Information

Description of Additional Supplementary Files

Supplementary Movie 1

Supplementary Movie 2

Reporting Summary

## Data Availability

The source data for building the tables and figures in this work are provided in this paper. The original BCP data are accessible online (https://nda.nih.gov/edit_collection.html?id=2848). The 4D infant brain volumetric atlas used in this work is also publicly available (https://www.nitrc.org/projects/uncbcp_4d_atlas/). Other data supporting this study’s findings are available from the corresponding author. Source data are provided with this paper.

## Source data

Source data

## Code availability

Processing of MR images was performed using iBEAT V2.0 (http://www.ibeat.cloud/). Subcortical segmentation was performed using our infant-dedicated subcortical segmentation method (10.1016/j.neuroimage.2023.119931). Image registration was implemented using Advanced Normalization Tools (ANTs 2.2.0, https://github.com/ANTsX/ANTs). The GAMM used in the statistical analysis was conducted in R 3.5.0 (primarily packages: gam 1.22-2, https://cran.r-project.org/web/packages/gam/index.html). Curve figures were made using Matlab 2021b and Microsoft Excel 2021. The surface meshes were visualized with ParaView 5.8.0 (https://www.paraview.org/). The manual correction was performed using ITK-SNAP 3.8.0 (http://www.itksnap.org/). Other codes or software used in this study are available from the corresponding author.
