# Supplementary material for: Four-dimensional mapping of dynamic longitudinal brain subcortical development and early learning functions in infants
Source: Nat Commun. 2023 Jun 22;14:3727. doi: 10.1038/s41467-023-38974-9 (PMC10287661; doi:10.1038/s41467-023-38974-9)
Supplement: Supplementary file 1 — Supplementary Information [file 41467_2023_38974_MOESM1_ESM.pdf]

Supplementary Information

1. Inclusion and Exclusion Criteria

Inclusion criteria: 1) born from 37 to 42 weeks GA, 2) at an appropriate birth weight matching to the GA, and 3) free of major pregnancy and delivery complications. **Exclusion criteria**: 1) being adopted, 2) identified schizophrenia, autism, bipolar disorder, or intellectual disability, 3) having any medical or genetic conditions related to growth, development, or cognition, 4) having any MRI contraindication, 5) maternal alcohol or illicit drug use, placental abruption, maternal pre-eclampsia, and maternal HIV status during pregnancy. The recruitment flow chart and more details on enrolling inclusion and exclusion criteria can be found in [1].

2. Materials and Methods

Materials. Table S1 presents the details of the data used in this work. Of note, the BCP cohort doesn't include twins.

Table S1. Participant characteristics.

Characteristic	Data
**Total number of scans acquired**	702 scans
**Remaining number of scans after each step**	
1. **After quality control (excessive motion, insufficient coverage, and/or ghosting)**	633 scans
2. **After removing scans with missing T1w or T2w images**	564 scans
3. **After subcortical segmentation and manual correction**	513 scans
**Total number of subjects**	231 subjects (513 scans)
**Subjects having only 1 scan**	92 subjects
**Subjects having 2 scans**	65 subjects
**Subjects having ≥ 3 scans**	74 subjects
**Sex**	126 females / 105 males
**Age range**	10-809 days (scan age)

Fig. S1 exhibits the number of subjects per month with respect to sex (Female/Red, Male/Blue).

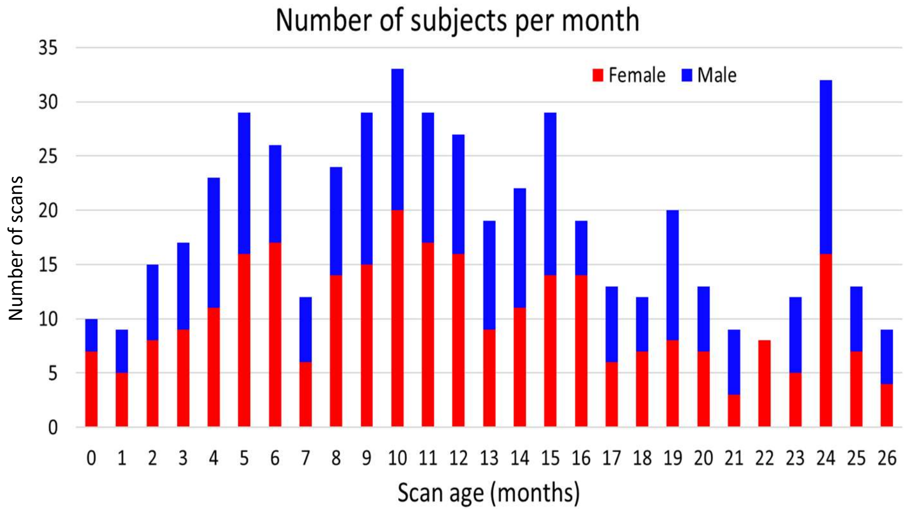

Fig. S1. The number of subjects at each scan age.

Subcortical segmentation and manual correction. We performed subcortical segmentation with our previous infant-dedicated deep learning-based subcortical segmentation method [3,4]. To cope with the dynamic appearance changes, low tissue contrast, and tiny subcortical size in infant brain MR images, our method is a context-guided (signed distance maps (SDMs)), attention-based, and coarse-to-fine learning deep framework. In detail, at the coarse stage, we proposed an SDM-Unet to directly predict the SDMs from multi-modal intensity images, including T1w, T2w, and the ratio of T1w and T2w images, which can leverage the spatial context information, including the structural position information and the shape information of the target structure, to generate high-quality SDMs. At the fine stage, we further proposed a multi-source and multi-path attention Unet (M2A-Unet). Then, the SDMs predicted by SDM-Unet, which encode spatial-context information of each subcortical structure, are integrated with the multi-modal intensity images as the input of M2A-Unet for achieving refined segmentation. Besides, both the 3D spatial and channel attention blocks are added to guide the M2A-Unet to focus more on the important subregions and channels. Due to the significantly different appearances of the infant brain MR images across ages, we manually delineated 48 scans within four representative age ranges, i.e., 0M-3M, 6M, 9M-12M, and 18M-24M, and each age range has 12 scans. We then separately trained a deep network for each age group. A stratified 6-fold cross-validation strategy is employed, and each fold consists of 10 training images and 2 testing images.

In **Fig. S2**, we illustrated subcortical segmentation results (overlaid on T1w images) of one subject with 4 longitudinal scans. The first 3 rows are the typical slices from axial, sagittal, and coronal views. The last row shows the mesh surfaces. We can find that the segmentation results achieved by our method have high accuracy with precisely matched boundaries. The edges of each subcortical structure are also very smooth and free of over-shootings.

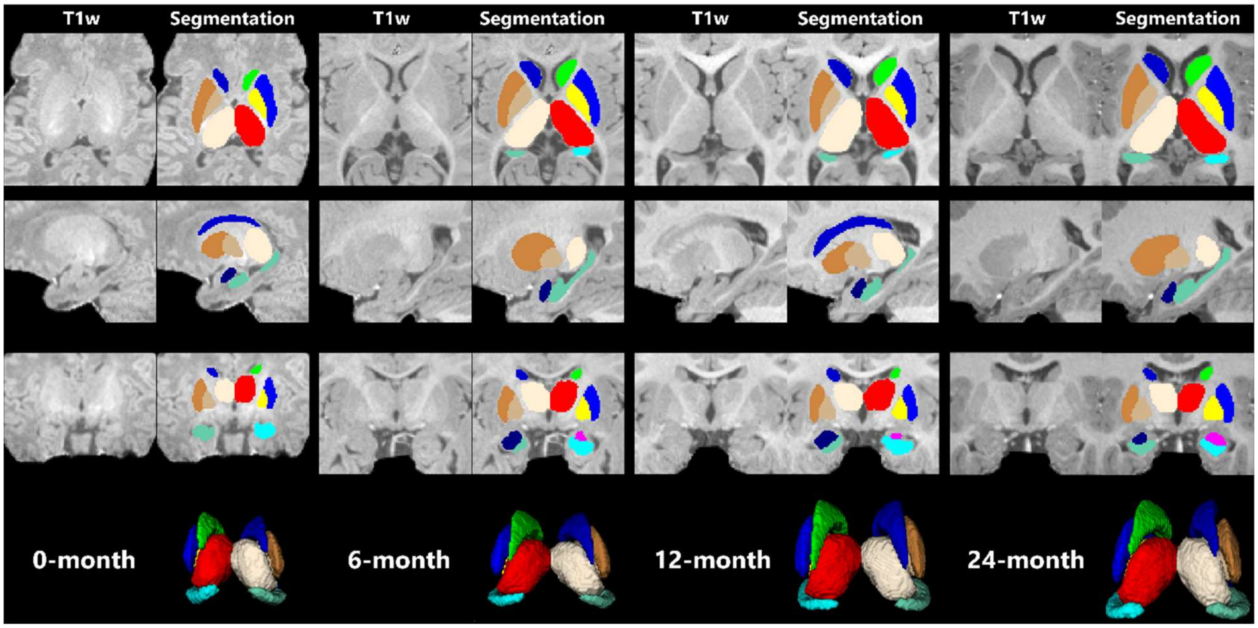

Fig. S2. The T1w images and segmentation results of the 12 subcortical structures from one subject with 4 longitudinal scans.

In **Table S2** and **Table S3**, we respectively present the segmentation accuracy in terms of the Dice similarity coefficient (DSC) and average symmetric surface distance (ASSD) (mean and standard deviation). Our method can achieve high DSC value (mean DSC value higher than 0.92) across all subcortical structures. Each of the achieved subcortical segmentation maps was further manually checked by one expert to guarantee segmentation consistency and the segmentation maps with minor errors were corrected during checking. Finally, 513 out of 564 scans survived the manual check. All the remaining subcortical segmentation maps have high accuracy, which can guarantee the accuracy of volumetric analysis.

Table S2. The DSC values of the automatic segmentation of 12 subcortical structures in each age group.

DSC	0M-3M	6M	12M	18M-24M
Thalamus_L	0.9447 ± 0.0095	0.9604 ± 0.0157	0.9660 ± 0.0089	0.9661 ± 0.0147
Caudate_L	0.9087 ± 0.0154	0.9421 ± 0.0187	0.9482 ± 0.0112	0.9500 ± 0.0145
Putamen_L	0.9167 ± 0.0172	0.9344 ± 0.0185	0.9534 ± 0.0135	0.9521 ± 0.0170
Pallidum_L	0.8606 ± 0.0310	0.9195 ± 0.0152	0.9518 ± 0.0097	0.9488 ± 0.0161
Hippocampus_L	0.8844 ± 0.0283	0.9029 ± 0.0178	0.9182 ± 0.0166	0.9217 ± 0.0131
Amygdala_L	0.8686 ± 0.0318	0.8616 ± 0.0266	0.8764 ± 0.0378	0.8970 ± 0.0210
Thalamus_R	0.9498 ± 0.0103	0.9615 ± 0.0141	0.9599 ± 0.0101	0.9650 ± 0.0069
Caudate_R	0.9095 ± 0.0162	0.9389 ± 0.0199	0.9536 ± 0.0109	0.9521 ± 0.0116
Putamen_R	0.9176 ± 0.0147	0.9336 ± 0.0114	0.9511 ± 0.0121	0.9442 ± 0.0131
Pallidum_R	0.8549 ± 0.0223	0.9129 ± 0.0240	0.9476 ± 0.0117	0.9293 ± 0.0106
Hippocampus_R	0.8817 ± 0.0157	0.9065 ± 0.0218	0.9010 ± 0.0269	0.9067 ± 0.0186

Amygdala_R	0.8550 ± 0.0557	0.8675 ± 0.0291	0.8896 ± 0.0243	0.8941 ± 0.0160

Table S3. The ASSD values (mm) of the automatic segmentation of 12 subcortical structures in each age group.

ASSD	0M-3M	6M	12M	18M-24M
Thalamus_L	0.067 ± 0.014	0.071 ± 0.015	0.73 ± 0.017	0.079 ± 0.023
Caudate_L	0.025 ± 0.008	0.037 ± 0.010	0.039 ± 0.012	0.044 ± 0.015
Putamen_L	0.082 ± 0.012	0.095 ± 0.016	0.099 ± 0.017	0.103 ± 0.019
Pallidum_L	0.048 ± 0.017	0.053 ± 0.017	0.061 ± 0.019	0.070 ± 0.024
Hippocampus_L	0.050 ± 0.010	0.059 ± 0.013	0.063 ± 0.017	0.70 ± 0.022
Amygdala_L	0.051 ± 0.013	0.057 ± 0.014	0.062 ± 0.018	0.068 ± 0.020
Thalamus_R	0.063 ± 0.011	0.064 ± 0.010	0.078 ± 0.016	0.083 ± 0.019
Caudate_R	0.032 ± 0.006	0.036 ± 0.08	0.041 ± 0.010	0.051 ± 0.013
Putamen_R	0.063 ± 0.022	0.070 ± 0.023	0.076 ± 0.025	0.079 ± 0.029
Pallidum_R	0.105 ± 0.036	0.117 ± 0.039	0.138 ± 0.042	0.143 ± 0.048
Hippocampus_R	0.056 ± 0.010	0.055 ± 0.011	0.062 ± 0.013	0.069 ± 0.016
Amygdala_R	0.053 ± 0.014	0.061 ± 0.016	0.069 ± 0.017	0.074 ± 0.021

Subcortical surfaces mapping. Fig. S3 shows the flowchart for subcortical surfaces mapping. The following details the major steps.

1) We exploited the T1w, T2w, and the reliable tissue probability maps (obtained by our iBeat V2.0 Cloud pipeline) as input to construct a high-quality 4D infant brain volumetric atlas based on the BCP dataset using the SyGN template construction method from ANTs. This infant-dedicated brain atlas includes densely sampled time points (i.e., 0, 1, 2, 3, 4, 5, 6, 7, 8, 9, 10, 11, 12, 15, 18, 21, and 24 months of age). During the atlas construction procedure, we established the deformations between each age-specific atlas and the age-matched individual scans.

2) We established the voxel-wise anatomical deformations across atlases at different time points. Note that to achieve more accurate registration, it is better to regard the image with high-information as the target, and warp the low-information image to the high-information image. Therefore, to obtain the aforementioned deformations, we performed the registration sequentially in an age-increasing manner.

3) We reconstructed surface mesh representations of each subcortical structure for the 0-month atlas as the initial reference surfaces.

4) We warped the subcortical surface mesh representations of the 0-month atlas to individual scans by exploiting both the established anatomical deformations across age-specific atlases and the deformations between the age-specific atlases and individual scans following an age-increasing manner. Note the established deformations across age-specific atlases and the deformations between each age-specific atlas and corresponding individual scans are combined into one transformation to warp the surface mesh representations.

5) We calculated the local surface area of each vertex on each individual subcortical surface to fit the vertex-wise developmental trajectories.

By using the reliable tissue probability maps (TPMs) as extra information in registration, the dynamic appearance changes and low contrast problems in infant brain MR images can be well handled, leading to accurate registration. Consequently, our 4D atlas has high quality with sharp structural patterns and the achieved anatomical deformations are also of high accuracy, leading to accurate subcortical surface mapping.

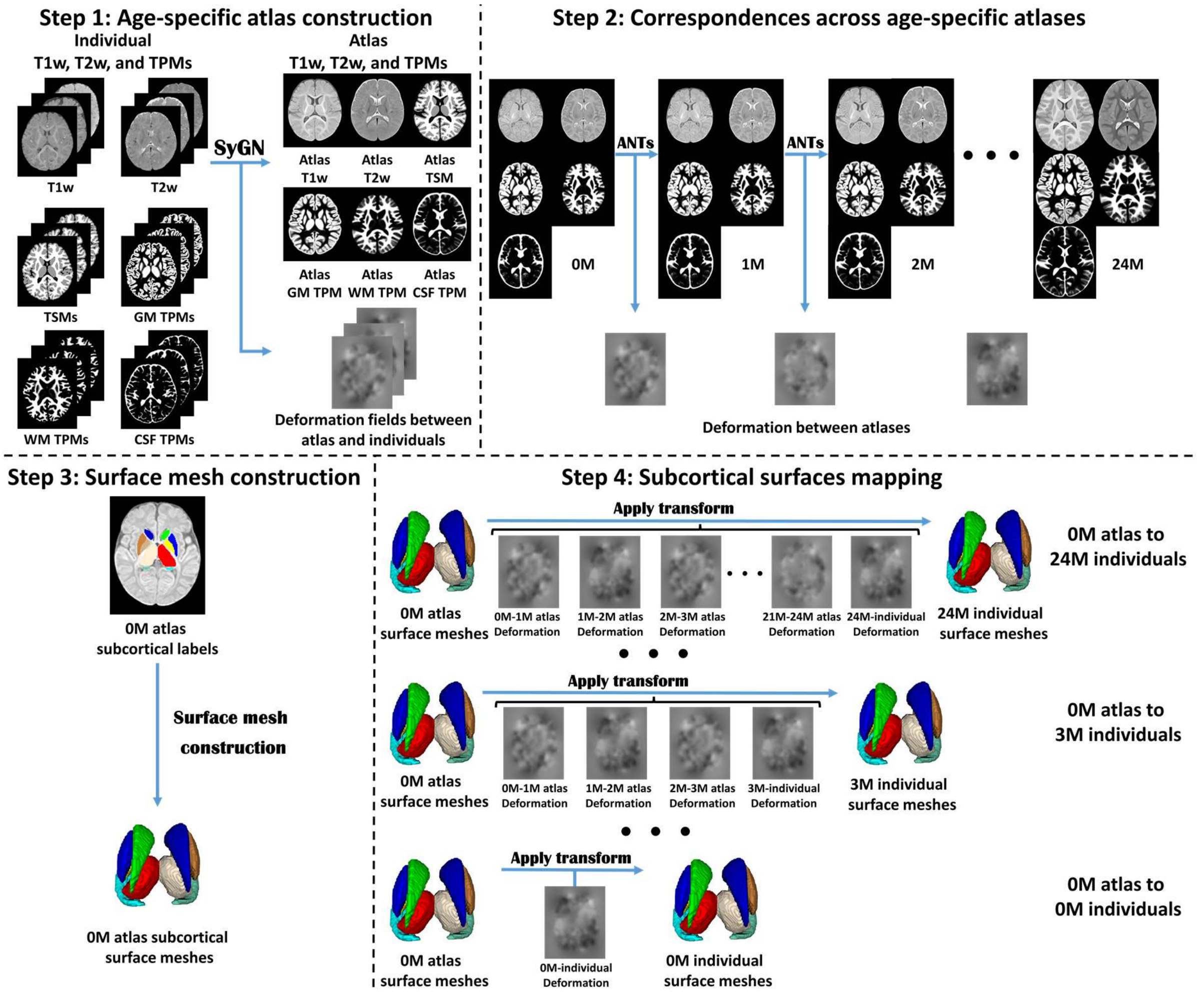

Fig. S3. Flowchart for subcortical surfaces mapping. M: Month. TPM: tissue pobabilty map. TSM: tissue segmentation map. GM: gray matter; WM: white matter; CSF: cerebrospinal fluid.

In **Table S4**, we presented the registration accuracy from the 0-month atlas to each individual scan within different age ranges based on the deformation fields across age-specific atlases and those between the age-specific atlases and individual scans. To quantitively evaluate the registration accuracy, we used the concatenated deformation field to warp the subcortical surfaces of 0-month atlas to each individual scan and calculate the ASSD with respect to the

surfaces of the manually corrected individual subcortical labels. The ASSD values are around 0.1 mm, indicating the high accuracy of surface mapping.

Table S4. The ASSD values (in mm) of the 12 subcortical structures of each age group by registration.

ASSD	0M-3M	6M	12M	18M-24M
Thalamus_L	0.187 ± 0.036	0.097 ± 0.016	0.104 ± 0.017	0.153 ± 0.029
Caudate_L	0.063 ± 0.012	0.041 ± 0.007	0.035 ± 0.004	0.028 ± 0.006
Putamen_L	0.113 ± 0.019	0.107 ± 0.018	0.069 ± 0.011	0.077 ± 0.016
Pallidum_L	0.068 ± 0.014	0.061 ± 0.005	0.043 ± 0.008	0.048 ± 0.005
Hippocampus_L	0.099 ± 0.010	0.081 ± 0.019	0.075 ± 0.015	0.101 ± 0.037
Amygdala_L	0.071 ± 0.008	0.057 ± 0.009	0.089 ± 0.017	0.089 ± 0.015
Thalamus_R	0.053 ± 0.005	0.085 ± 0.011	0.047 ± 0.006	0.052 ± 0.006
Caudate_R	0.031 ± 0.003	0.036 ± 0.010	0.044 ± 0.007	0.024 ± 0.003
Putamen_R	0.082 ± 0.008	0.095 ± 0.013	0.048 ± 0.005	0.061 ± 0.013
Pallidum_R	0.102 ± 0.016	0.070 ± 0.004	0.036 ± 0.005	0.055 ± 0.007
Hippocampus_R	0.078 ± 0.016	0.065 ± 0.015	0.058 ± 0.010	0.071 ± 0.018
Amygdala_R	0.062 ± 0.011	0.067 ± 0.010	0.071 ± 0.021	0.051 ± 0.003

The t-test comparisons. For identification of high-/low-growth regions, the t-test statistic is calculated as follows:

$$T_2(s,t) = \frac{\hat{r}(s,t) - \hat{r}_m(t)}{\sqrt{\widehat{Var}[\hat{r}(s,t)] + \widehat{Var}[\hat{r}_m(t)]}}, \qquad \forall s \in S, t \in T = \{30,60,\dots,810\}; \qquad (1)$$

where S indicates the set of vertices of a subcortical structure, $\hat{r}(s,t)$ is the estimated growth rate on vertex *s* at time *t* estimated from the derivative of model (b), $\hat{r}_m(t)$ is the median of $\{\hat{r}(s,t): s \in S\}$. For low-growth region identification, p-values are calculated as $p_l(s,t) = F_{df}\big(T_2(s,t)\big)$, where $F_{df}(\cdot)$ is the cummulative distribution function of the *t* distribution with degree of freedom *df* = *n*-*p*, where *n* is the number of observations and *p* is the model degree of freedom. The variance of $[\hat{r}(s,t)]$ is estimated from the GAMM model, while the variance of $[\hat{r}_m(t)]$ is approximately zero from the large sample property. To adjust for multiple comparisons based on false discovery rate (FDR), q-values are calculated based on $\{p_l(s,t): s \in S, t \in T\}$ to control for the number of vertices and the 27 time points (months) for the target subcortical structure. For high-growth region identification, the same procedure is adopted except for *P*-values $p_h(s,t) = 1 - F_{df}\big(T_2(s,t)\big)$, because larger $T_2(s,t)$ are considered more significant. The t-statistics with q-value < 0.05 are shown in **Fig. 4**.

The stratified bootstrap. We apply the stratified bootstrapping in [2] to obtain *P*-values for volumetric changes demonstrated in **Table 1**. Specifically, for longitudinal data with repeated measurements of subjects, the unit of sampling is no longer the particular observation time point-level but the subject-level; this is because otherwise the observation-level resampling will break the correlation structure of the data between different observations and distort the

resultant distributions. Subjects can be divided into 4 categories: with 1 visit, 2 visits, 3 visits and ≥ 4 visits; to keep the sample size of both the observations and subjects in the resampled data the same as original data, for each category of subjects we resampled separately with the same sample size as the real data, and then combined the resampled observations from the 4 categories together. This will not only generate the resampling data with the same number of subjects and observations in each category, but also keep the subject-level correlation structure. Finally, the difference $\left|\hat{Y}(t_2) - \hat{Y}(t_1)\right|$ were estimated 1000 times based on 1000 resampled datasets and the bootstrapped standard deviation (*SD*) of $\hat{Y}(t_2) - \hat{Y}(t_1)$ was estimated, where $\hat{Y}(t_2)$ and $\hat{Y}(t_1)$ are fitted values of the trajectories $Y(t)$ at the age $t = t_2$ and t_1, respectively. The Z-statistics $Z(t_1, t_2) = \left|\hat{Y}(t_2) - \hat{Y}(t_1)\right| / SD[\hat{Y}(t_2) - \hat{Y}(t_1)]$ was used to calculated the p-values to test difference between $Y(t_2)$ and $Y(t_1)$.

Analyzing the sex effect of area expansion. Similar to the procedures in plotting **Fig. 3**, we applied the GAMM model to fit the expansion rate trajectories of surface area for each vertex with respect to males and females. Then, for each vertex, we compared the values between males and females and performed a t-test to discover the sex differences on the expansion rate of surface area. The FDR-adjusted *P*-values show no significant sex differences in the rate of area expansion for each subcortical structure within the age range examined.

3. *P*-values for volumetric changes of each subcortical structure during different age ranges:

Table S5. Volumetric growth rates of the left subcortical structures at different developmental stages during the first 2 postnatal years. M: Month. Values in shadow indicate $p < 0.05$ after Bonferroni Correction. We use zero to indicate $p <$ 1E-16. (Corresponding to **Table 1**).

Age range	Thalamus (Male/Female)	Caudate (Male/Female)	Putamen (Male/Female)	Pallidum (Male/Female)	Hippocampus (Male/Female)	Amygdala (Male/Female)
0M-3M	→0/→0	→0/→0	→0/→0	→0/→0	→0/→0	1E-14/→0
3M-6M	→0/→0	→0/→0	→0/→0	→0/→0	→0/→0	→0/→0
6M-9M	0.07/0.11	→0/→0	→0/→0	1E-14/5E-11	→0/→0	→0/→0
9M-12M	0.2/0.15	→0/→0	→0/→0	2E-07/3E-05	→0/→0	→0/→0
12M-18M	→0/→0	1E-12/1E-11	→0/→0	→0/→0	→0/→0	→0/→0
18M-24M	→0/→0	2E-08/8E-10	→0/→0	→0/1.55E-12	8E-11/8E-13	5E-09/→0
0M-6M	→0/→0	→0/→0	→0/→0	→0/→0	→0/→0	→0/→0
0M-12M	→0/→0	→0/→0	→0/→0	→0/→0	→0/→0	→0/→0
0M-24M	→0/→0	→0/→0	→0/→0	→0/→0	→0/→0	→0/→0

Table S6. Volumetric growth rates of the right subcortical structures at different developmental stages during the first 2 postnatal years. M: Month. Values in shadow indicate $p < 0.05$ after Bonferroni Correction. We use zero to indicate $p <$ 1E-16. (Corresponding to **Table 2**).

Age range	Thalamus (Male/Female)	Caudate (Male/Female)	Putamen (Male/Female)	Pallidum (Male/Female)	Hippocampus (Male/Female)	Amygdala (Male/Female)
0M-3M	→0/→0	→0/→0	→0/→0	→0/→0	→0/→0	1E-14/→0
3M-6M	→0/→0	→0/→0	→0/→0	→0/→0	→0/→0	→0/→0
6M-9M	0.09/0.08	→0/→0	→0/→0	4E-4/1E-3	→0/→0	→0/→0
9M-12M	0.31/0.23	→0/→0	→0/→0	7E-06/1E-4	→0/→0	→0/→0
12M-18M	→0/→0	2E-13/→0	→0/→0	→0/→0	→0/→0	→0/→0
18M-24M	→0/→0	3E-05/1E-05	1E-11/1E-08	2E-12/1E-11	→0/→0	1E-13/→0
0M-6M	→0/→0	→0/→0	→0/→0	→0/→0	→0/→0	→0/→0
0M-12M	→0/→0	→0/→0	→0/→0	→0/→0	→0/→0	→0/→0
0M-24M	→0/→0	→0/→0	→0/→0	→0/→0	→0/→0	→0/→0

4. Detailed results for multiple regression:

1) FM~Time+Gender+Site+Income+Edu+ICV+Putamen +Caudate +(1|ID)

	Estimate	Std. Error	df	t-value	Pr(>|t|)
(Intercept)	53.5253	0.5948	129.8996	89.9921	0
Time	-3.0933	0.9451	292.5256	-3.2731	0.0012
Gender	-1.1218	0.6611	137.9076	-1.6970	0.0920
Site	-0.6247	0.6250	121.4241	-0.9994	0.3196
Income	-0.3267	0.6373	133.8496	-0.5126	0.6091
Edu	0.3823	0.627	148.2852	0.6097	0.543
ICV	4.4964	0.9961	226.1698	4.5138	0
Putamen	1.5392	0.7177	228.7383	2.1446	0.0330
Caudate	1.1909	0.7036	174.1231	1.6927	0.0923

2) RL~Time+Gender+Site+Income+Edu+ICV+ Thalamus+(1|ID)

	Estimate	Std. Error	df	t-value	Pr(>|t|)
(Intercept)	50.7994	0.6188	136.7478	82.0988	0
Time	2.4011	0.8809	284.9548	2.7258	0.0068
Gender	-1.9722	0.6900	145.0272	-2.8584	0.0049
Site	1.0666	0.6506	127.6463	1.6395	0.1036
Income	0.5445	0.6569	136.4646	0.8288	0.4087
Edu	-0.8613	0.6489	151.8595	-1.3273	0.1864
ICV	1.7259	1.3010	152.2223	1.3266	0.1866
Thalamus	3.9588	0.9995	244.2239	3.9607	0.0001

3) EL~Time+Gender+Site+Income+Edu+ICV+ Thalamus+(1|ID)

	Estimate	Std. Error	df	t-value	Pr(>|t|)
(Intercept)	51.5413	0.5920	176.6591	87.0649	0
Time	0.4751	0.8191	304.8055	0.5801	0.5623
Gender	-0.4768	0.6586	186.7925	-0.7240	0.4711
Site	1.0278	0.6237	169.6478	1.6479	0.1012

Income	0.0771	0.6285	176.5660	0.1226	0.9025
Edu	-0.5777	0.6187	189.7008	-0.9336	0.3517
ICV	0.2404	1.2384	203.6449	0.1941	0.8463
Thalamus	3.0932	0.9352	283.3845	3.3077	0.0011

5. Asymmetry Index Trajectories of Subcortical Volumes

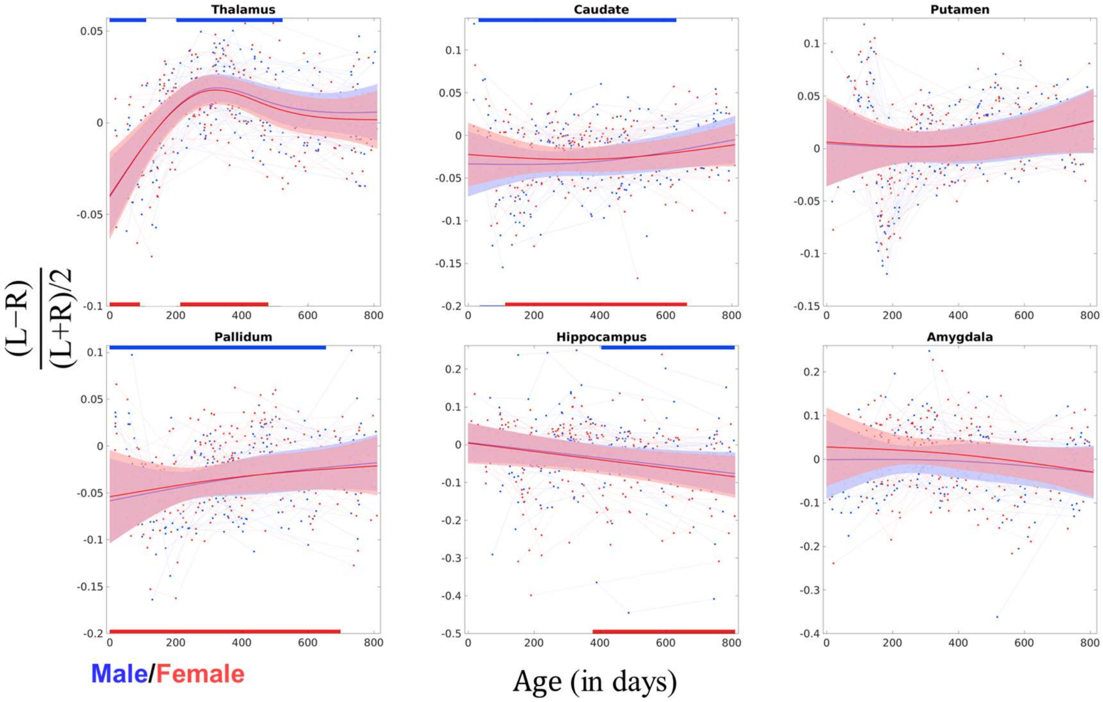

Fig. S4. Asymmetry index trajectories of different subcortical structures. The values (including 95% confidence intervals) higher than 0 mean leftward asymmetries (larger sizes of left structures), and vice versa. The red and blue horizontal bars respectively exhibit the statistically significant volumetric differences between the left and right structures for females and males.

From **Fig. S4**, we can find that the putamen and amygdala don't exhibit statistically significant asymmetry in both females and males during the age range examined. The right caudate, pallidum, and hippocampus have significantly larger volumes than the left ones during different age ranges, while the left thalamus is significantly smaller than the right thalamus during the first three months, gradually becomes significantly larger than the right thalamus from around 7M to 18M, and then the asymmetry disappears. Studies on datasets with larger sample sizes covering larger age ranges are desired for further verifying the asymmetry of each subcortical structure during early development.

6. Trajectories of Early Subcortical Volume Development without adjusting ICV

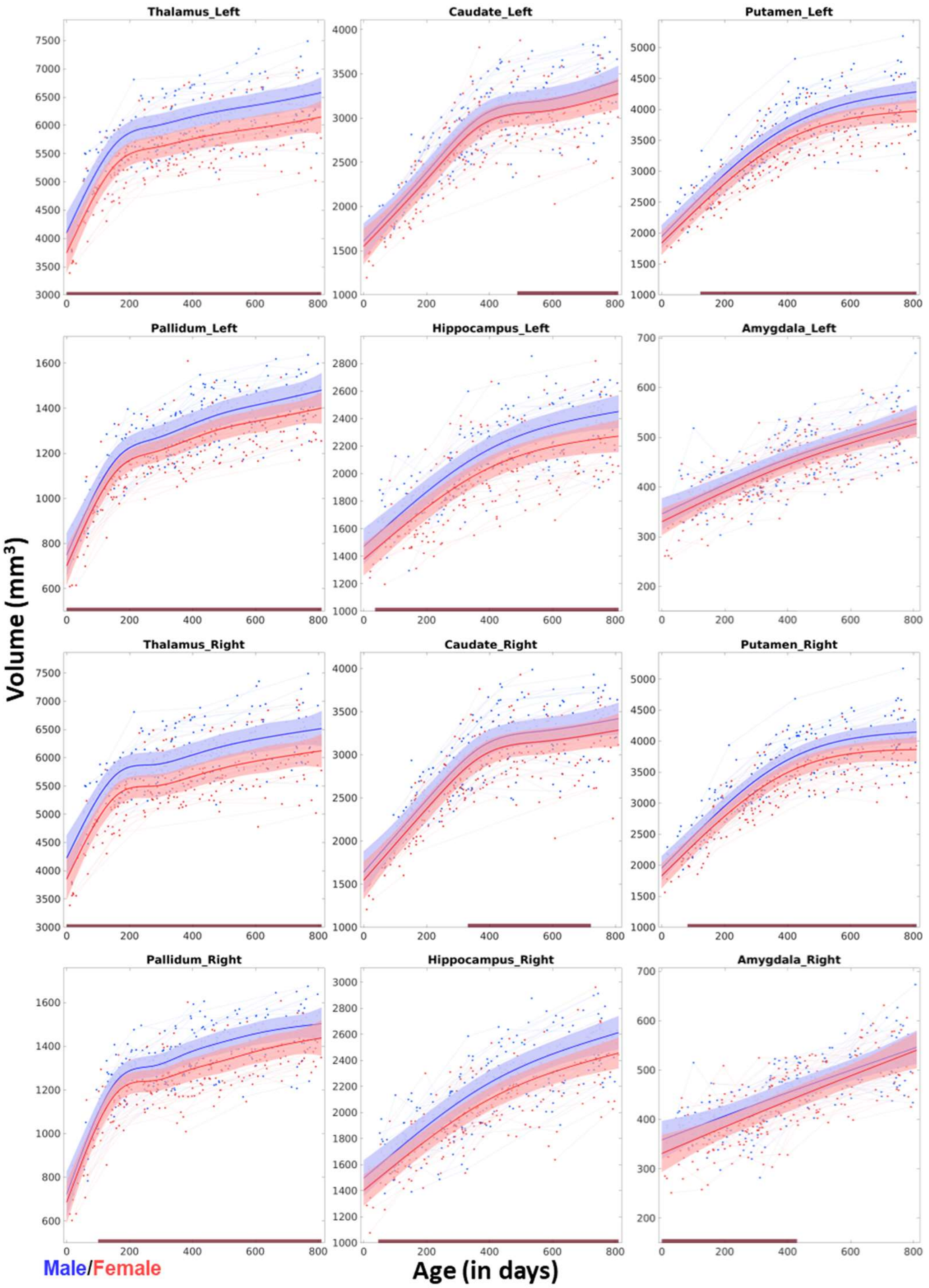

Fig. S5. Longitudinal volumetric developmental trajectories of different subcortical structures without adjusting ICV. Plots show both the individual-level (thin lines) and fitted population-level (solid curves) developmental trajectories of the bilateral volumes of the thalamus, caudate, putamen, pallidum, hippocampus, and amygdala structures. The shaded ribbon around each curve denotes 95% confidence intervals.

Supplementary references:

[1] Howell, Brittany R., et al. "The UNC/UMN Baby Connectome Project (BCP): An overview of the study design and protocol development." NeuroImage 185 (2019): 891-905.

[2] Sherman, Michael, and Saskia le Cessie. "A comparison between bootstrap methods and generalized estimating equations for correlated outcomes in generalized linear models." Communications in Statistics-Simulation and Computation 26.3 (1997): 901-925.

[3] Chen, Liangjun, et al. "A deep spatial context guided framework for infant brain subcortical segmentation." Medical Image Computing and Computer Assisted Intervention–MICCAI 2020: 23rd International Conference, Lima, Peru, October 4–8, 2020, Proceedings, Part VII 23. Springer International Publishing, 2020.

[4] Chen, Liangjun, et al. "An attention-based context-informed deep framework for infant brain subcortical segmentation." NeuroImage 269 (2023): 119931.
