# Supplementary material for: Four-dimensional mapping of dynamic longitudinal brain subcortical development and early learning functions in infants
Source: Nat Commun. 2023 Jun 22;14:3727. doi: 10.1038/s41467-023-38974-9 (PMC10287661; doi:10.1038/s41467-023-38974-9)
Supplement: Supplementary file 2 — Description of Additional Supplementary Files [file 41467_2023_38974_MOESM2_ESM.pdf]

Description of Additional Supplementary Files

File Name: Supplementary Movie 1.

Description: Dynamic surface area expansion rate map of the infant subcortex from birth to 24 months of age. The thalamus, caudate, putamen, pallidum, hippocampus, and amygdala are separately assembled in anatomical space and shown in rotation.

File Name: Supplementary Movie 2.

Description: Dynamic surface area expansion rate map of the infant subcortex during the first two years shown in 4 views.
