# Supplementary material for: Four-dimensional mapping of dynamic longitudinal brain subcortical development and early learning functions in infants
Source: Nat Commun. 2023 Jun 22;14:3727. doi: 10.1038/s41467-023-38974-9 (PMC10287661; doi:10.1038/s41467-023-38974-9)
Supplement: Supplementary file 5 — Reporting Summary [file 41467_2023_38974_MOESM5_ESM.pdf]

nature portfolio

Corresponding author(s): Gang Li
Last updated by author(s): 2023-2-23

Reporting Summary

Nature Portfolio wishes to improve the reproducibility of the work that we publish. This form provides structure for consistency and transparency in reporting. For further information on Nature Portfolio policies, see our Editorial Policies and the Editorial Policy Checklist.

Statistics

For all statistical analyses, confirm that the following items are present in the figure legend, table legend, main text, or Methods section.

n/a	Confirmed	
☐	☒	The exact sample size (*n*) for each experimental group/condition, given as a discrete number and unit of measurement
☐	☒	A statement on whether measurements were taken from distinct samples or whether the same sample was measured repeatedly
☐	☒	The statistical test(s) used AND whether they are one- or two-sided<br>*Only common tests should be described solely by name; describe more complex techniques in the Methods section.*
☐	☒	A description of all covariates tested
☐	☒	A description of any assumptions or corrections, such as tests of normality and adjustment for multiple comparisons
☐	☒	A full description of the statistical parameters including central tendency (e.g. means) or other basic estimates (e.g. regression coefficient) AND variation (e.g. standard deviation) or associated estimates of uncertainty (e.g. confidence intervals)
☐	☒	For null hypothesis testing, the test statistic (e.g. *F*, *t*, *r*) with confidence intervals, effect sizes, degrees of freedom and *P* value noted<br>*Give P values as exact values whenever suitable.*
☒	☐	For Bayesian analysis, information on the choice of priors and Markov chain Monte Carlo settings
☒	☐	For hierarchical and complex designs, identification of the appropriate level for tests and full reporting of outcomes
☐	☒	Estimates of effect sizes (e.g. Cohen's *d*, Pearson's *r*), indicating how they were calculated

Our web collection on statistics for biologists contains articles on many of the points above.

Software and code

Policy information about availability of computer code

Data collection	No software was used.
Data analysis	Processing of MR images was performed using iBEAT V2.0 (http://www.ibeat.cloud/). Subcortical segmentation was performed using our infant-dedicated subcortical segmentation method (https://doi.org/10.1016/j.neuroimage.2023.119931). Image registration was implemented using Advanced Normalization Tools (ANTs 2.2.0, https://github.com/ANTsX/ANTs). The GAMM used in the statistic analysis was conducted in R 3.5.0 (primarily packages: gam 1.22-2, https://cran.r-project.org/web/packages/gam/index.html). Curve figures were made using Matlab 2021b and Microsoft Excel 2021. The surface meshes were visualized with ParaView 5.8.0 (https://www.paraview.org/). The manual correction was performed using ITK-SNAP 3.8.0 (http://www.itksnap.org/). Other codes or software used in this study are available from the corresponding author.

For manuscripts utilizing custom algorithms or software that are central to the research but not yet described in published literature, software must be made available to editors and reviewers. We strongly encourage code deposition in a community repository (e.g. GitHub). See the Nature Portfolio guidelines for submitting code & software for further information.

Data

Policy information about availability of data

All manuscripts must include a data availability statement. This statement should provide the following information, where applicable:

- Accession codes, unique identifiers, or web links for publicly available datasets
- A description of any restrictions on data availability
- For clinical datasets or third party data, please ensure that the statement adheres to our policy

The source data are provided with this paper. The original BCP data are accessible online (https://nda.nih.gov/edit_collection.html?id=2848) with the NIH's permission. The 4D infant brain volumetric atlas is also publicly available (https://www.nitrc.org/projects/uncbcp_4d_atlas/). Other data supporting this study's findings are also available for subject who has the NIH's permission of accessing the original BCP data from the corresponding author.

Human research participants

Policy information about studies involving human research participants and Sex and Gender in Research.

Reporting on sex and gender	The breakdown of the BCP participant sample by biological sex was 54.5% female (total of 231 subjects with 126 females and 105 males). Biological sex was included as a covariate when running the GAMM-related analyses and the Mullen score-related behavior studies. Results of the gross volumetric development of each subcortical structure are split into sex-specific analyses, and sexual differences of the gross volumetric developmental patterns are reported.
Population characteristics	The age range of the subjects in this study is between birth and 27 months of age. All the subjects in this study are normal under following criteria. They are eligible if they 1) were born at a gestational age of 37–42 weeks, 2) had a birth weight appropriate for gestational age, and 3) had an absence of major pregnancy and delivery complications. Children are excluded from the BCP if they were born prior to 37 weeks gestation, had a birth weight lower than 2,000 grams, or if they had any major delivery complications. Major delivery complications may include neonatal hypoxia or neonatal illness requiring a greater than two day NICU stay. They are also excluded if they: 1) are adopted, 2) have a first degree relative with autism, intellectual disability, schizophrenia, or bipolar disorder, 3) have any significant medical and/or genetic conditions affecting growth, development, or cognition, or 4) have any contraindication to MRI. Additional exclusion criteria include major pre- and/or perinatal issues including: maternal pre-eclampsia, placental abruption, maternal HIV status, and maternal alcohol or illicit drug use during pregnancy. Finally, children are excluded from the study if their caregivers are unable to communicate in English at a level to provide informed consent. We refer to the original BCP paper (Howell et al., 2019) for further details.
Recruitment	Participants are recruited from existing registries at UNC and UMN based on state-wide birth records as well as from broader community resources (e.g., community centers and targeted day-care centers) to ensure the sample approximates the racial/ ethnic and socio-economic diversity of the US census. To augment recruitment of the youngest cohort of participants, we recruited participants prenatally by approaching expectant and new mothers at "The Birthplace" at UMN and the UNC Hospitals Newborn Nursery. Parents of all participants provided permission and informed consent prior to participation.
Ethics oversight	This study was approved by the Institutional Review Board at the University of North Carolina (UNC) at Chapel Hill, School of Medicine.

Note that full information on the approval of the study protocol must also be provided in the manuscript.

Field-specific reporting

Please select the one below that is the best fit for your research. If you are not sure, read the appropriate sections before making your selection.

[x] Life sciences [ ] Behavioural & social sciences [ ] Ecological, evolutionary & environmental sciences

For a reference copy of the document with all sections, see nature.com/documents/nr-reporting-summary-flat.pdf

Life sciences study design

All studies must disclose on these points even when the disclosure is negative.

Sample size	We analyzed 513 longitudinal scans from the BCP dataset, which passed quality control after subcortical segmentation and registration (both T1w and T2w MR images). The dataset included 231 subjects (126 females; 105 males), with varying numbers of scans for each subject. Figure S1 displays the distribution of scans across the first 26 postnatal months, illustrating a broadly balanced representation. During image processing, we retained as many scans as possible.<br>We validated that the sample size is sufficient for association analyses between subcortical volumes and Mullen scales. Power analyses were conducted using the "simr" R package (version 1.0.7, https://github.com/pitakakariki/simr), performing 1,000 simulations with all parameters derived from the BCP data. Using the likelihood ratio test, we were able to detect the effect sizes (standardized coefficients) of Thalamus on RL, Thalamus on EL, Putamen on FM, and Caudate on FM, as shown in Table 3, at respective power levels of 0.95, 0.88, 0.58, and 0.49, considering a significance level of 0.05.<br>Although obtaining and processing longitudinal infant brain MR images is challenging, leading to limited numbers of scans in infant brain-

	related studies, our work includes the largest number of longitudinal scans densely covering the first 26 postnatal months of life. While we cannot guarantee that all reported results can be identified at a power > 80%, many of findings can be further validated by existing literature.
Data exclusions	1) being adopted, 2) having schizophrenia, autism, bipolar disorder, or intellectual disability, 3) having any medical or genetic conditions related to growth, development, or cognition, 4) having any MRI contraindication, 5) maternal alcohol or illicit drug use, placental abruption, maternal pre-eclampsia, and maternal HIV status during pregnancy, 6) images and subcortical segmentations cannot pass the experts' quality control (i.e., with motion artifacts or missing parts).
Replication	To further testify the discovered developmental patterns of each subcortical structure during infancy, we applied our infant-dedicated subcortical segmentation method on a new infant brain MRI dataset from the National Database of Autism Research (NDAR). Of note, NDAR dataset was acquired at three time points (i.e., 6M, 12M, and 24M) with a resolution of 1×1×1 mm3 using different scanners with different protocols, compared to the BCP dataset. In total, 235 scans of healthy subjects from the NDAR dataset are segmented, and each time point respectively includes the following number of scans: 6M: 95 scans (36 Females / 59 Males); 12M: 82 scans (31 Females / 51 Males); 24M: 58 scans (22 Females / 36 Males). Based on the achieved subcortical segmentation results, we respectively calculated the growth rates of NDAR scans during the age ranges of 6M-12M and 12M-24M with respect to sex. The results illustrated that overall growth rates of each subcortical structure during the 6M-12M and 12M-24M are similar on both BCP and NDAR datasets. While, with the limited time points and without Mullen scales for the NDAR dataset, the full replication was not available for now.
Randomization	Participants are all term-born normative infants and were not allocated to experimental groups. Randomization was therefore not applicable.
Blinding	This study is an analysis of the longitudinal development of the normative infant subcortex. Participants are all term-born normative infants and were not allocated to experimental groups during recruitment, data processing and/or statistical analyses. Blinding was therefore not applicable.

Reporting for specific materials, systems and methods

We require information from authors about some types of materials, experimental systems and methods used in many studies. Here, indicate whether each material, system or method listed is relevant to your study. If you are not sure if a list item applies to your research, read the appropriate section before selecting a response.

Materials & experimental systems

n/a	Involved in the study
☒	☐ Antibodies
☒	☐ Eukaryotic cell lines
☒	☐ Palaeontology and archaeology
☒	☐ Animals and other organisms
☒	☐ Clinical data
☒	☐ Dual use research of concern

Methods

n/a	Involved in the study
☒	☐ ChIP-seq
☒	☐ Flow cytometry
☐	☒ MRI-based neuroimaging

Magnetic resonance imaging

Experimental design

Design type	Longitudinal structural MR image for studying the infant subcortical region development and related behavior development.
Design specifications	The MR images were collected using 3T Siemens Prisma MRI scanners from the recruited subjects during their age between 1 month and 27 months. The subcortical regions are then segmented and aligned to evaluate their developmental patterns.
Behavioral performance measures	The Mullen Scales of Early Learning (MSEL; Mullen, 1995) provides a standardized assessment of language, motor, and perceptual abilities for children of all ability levels through 5 years of age. The revised and updated version yields age-normed t scores, age equivalent scores, and percentile rankings for 5 subdomains: 1) gross motor, 2) fine motor, 3) visual reception, 4) receptive language, and 5) expressive language. Scores from the fine motor, visual reception, receptive language, and expressive language domains can be aggregated to yield an Early Learning Composite or developmental quotient value. It is also common to derive verbal (receptive language age equivalent score + expressive language age equivalent score/chronological age *100) and nonverbal (fine motor age equivalent score + visual reception age equivalent score/chronological age *100) developmental quotient scores from this assessment. The assessment takes between 20–45 minutes, depending on the age of the child. We implement the MSEL at every behavioral visit between 3 and 60 months of age. We refer to the original BCP paper (Howell et al., NeuroImage 2019) for further details.

Acquisition

Imaging type(s)	Structural MRI
Field strength	3T
Sequence & imaging parameters	T1w and T2w images have a spatial isotropic resolution of 0.8mm and were acquired with 208 sagittal slices using the following parameters, respectively: TR/TE = 2400/2.24 ms and TR/TE = 3200/564 ms.
Area of acquisition	Whole brain scan

Diffusion MRI ☐ Used ☒ Not used

Preprocessing

Preprocessing software	All images were preprocessed using the infant brain extraction and analysis toolbox (iBEAT V2.0 Cloud) (http://www.ibeat.cloud/). The N3 method was firstly performed on all images for intensity inhomogeneity correction with default parameters. For each subject, the T2w image was linearly aligned onto the corresponding T1w image using FLIRT v6.0 in FSL.
Normalization	All images are rigidly aligned using ANTs 2.2.0 (https://github.com/ANTsX/ANTs) to UNC-BCP 4D Infant Brain Volumetric Atlas
Normalization template	Our developed UNC-BCP 4D Infant Brain Volumetric Atlas (https://www.nitrc.org/projects/uncbcp_4d_atlas/) is used for normalization.
Noise and artifact removal	Incoming structural MRI data are assessed visually for excessive motion, insufficient coverage, and/or ghosting. Visual motion assessment is performed on a four-point scale (reject, major motion/borderline, minor motion/pass, excellent). Then bias-field correction is performed for the images with minor motion and excellent scale.
Volume censoring	Not applicable. Only structural MRI, involving T1-weighted and T2-weighted images, was used. Volumes were manually inspected and the scans were excluded if poor quality.

Statistical modeling & inference

Model type and settings	We utilized the generalized additive mixed models (GAMM) to fit the developmental trajectories of the total volume and each vertex's surface area of each subcortical structure. Two GAMMs were respectively used in our model fittings for the scenarios with or without considering gender. Multivariate analyses controlled for age, sex, and site. For the analysis between subcortical development and Mullen scores, we first calculated the logarithm of the subcortical-to-ICV (intracranial volume) ratio, and then carried out the association analyses between each subcortical volume ratio of the 6 subcortical structures and each of the 5 Mullen standardized t-scores based on linear mixed models, and the age, sex, site effect, maternal education, household income, and intracranial volume were controlled as confounding factors. For the area expansion analysis between caudate, putamen, and thalamus and Mullen scores, we applied the same mixed linear models to generate the associations between Mullen scores and each vertex. The achieved P-values are further adjusted by FDR correction. Finally, we carried out the vertex-wise areal behavior analysis at each vertex from the aligned surface. The functions and parameter setting were described in the manuscript and SI Materials and Methods.
Effect(s) tested	We tested the significant volumetric changes during different age ranges and revealed the statistically high-growth and low-growth rate regions for each subcortical structure. We also tested the relationships between subcortical development and Mullen scores and performed the area expansion analysis between caudate, putamen, and thalamus and Mullen scores.

Specify type of analysis: ☐ Whole brain ☒ ROI-based ☐ Both

Anatomical location(s)	Both T1w and T2w images were fed into our developed convolutional neural networks for infant brain subcortical segmentation (Chen et al, NeuroImage 2023), and each of the achieved subcortical segmentation maps was further manually-corrected by experts.
Statistic type for inference (See Eklund et al. 2016)	Vertex-wise statistic analysis is performed for revealing the statistically significant high-growth and low-growth regions of each subcortical structure. Cluster-wise statistic analysis is performed for exhibiting significant area changes, which used two nonparametric functions fitted with the cubic splines (smooth term setting as 4).
Correction	Multiple comparison correction using false discovery rate (FDR) with q=0.05 is performed for revealing the statistically significant high-growth and low-growth regions of each subcortical structure and all analyses related to Mullen scores.

Models & analysis

n/a	Involved in the study
☒	☐ Functional and/or effective connectivity
☒	☐ Graph analysis
☒	☐ Multivariate modeling or predictive analysis
